# Unexpected Rarity of the Pathogen *Batrachochytrium dendrobatidis* in Appalachian *Plethodon* Salamanders: 1957–2011

**DOI:** 10.1371/journal.pone.0103728

**Published:** 2014-08-01

**Authors:** Carly Muletz, Nicholas M. Caruso, Robert C. Fleischer, Roy W. McDiarmid, Karen R. Lips

**Affiliations:** 1 Department of Biology, University of Maryland, College Park, Maryland, United States of America; 2 Department of Biological Sciences, University of Alabama, Tuscaloosa, Alabama, United States of America; 3 Center for Conservation and Evolutionary Genetics, Smithsonian Conservation Biology Institute, National Zoological Park, Washington, DC, United States of America; 4 USGS Patuxent Wildlife Research Center, Smithsonian Institution, National Museum of Natural History, Washington, DC, United States of America; University of California-Riverside, United States of America

## Abstract

Widespread population declines in terrestrial *Plethodon* salamanders occurred by the 1980s throughout the Appalachian Mountains, the center of global salamander diversity, with no evident recovery. We tested the hypothesis that the historic introduction and spread of the pathogenic fungus *Batrachochytrium dendrobatidis* (*Bd*) into the eastern US was followed by *Plethodon* population declines. We expected to detect elevated prevalence of *Bd* prior to population declines as observed for Central American plethodontids. We tested 1,498 *Plethodon* salamanders of 12 species (892 museum specimens, 606 wild individuals) for the presence of *Bd*, and tested 94 of those for *Batrachochytrium salamandrivorans (Bs)* and for ranavirus. Field samples were collected in 2011 from 48 field sites across a 767 km transect. Historic samples from museum specimens were collected at five sites with the greatest number and longest duration of collection (1957–987), four of which were sampled in the field in 2011. None of the museum specimens were positive for *Bd*, but four *P. cinereus* from field surveys were positive. The overall *Bd* prevalence from 1957–2011 for 12 *Plethodon* species sampled across a 757 km transect was 0.2% (95% CI 0.1–0.7%). All 94 samples were negative for *Bs* and ranavirus. We conclude that known amphibian pathogens are unlikely causes for declines in these *Plethodon* populations. Furthermore, these exceptionally low levels of *Bd*, in a region known to harbor *Bd*, may indicate that *Plethodon* specific traits limit *Bd* infection.

## Introduction

Globally, amphibians have experienced population declines and extinctions [1], [2] with many recent population declines (e.g., [3], [4]) caused by *Batrachochytrium dendrobatidis* (*Bd*), a pathogenic fungus that causes chytridiomycosis [5]. Population declines of Central American plethodontid salamanders were documented [6] in the late 1970s to early 1980s by pairing field surveys with historic field notes and museum records; subsequent molecular analysis of these museum specimens [7] demonstrated that *Bd* was the likely driver of these declines.

The causes for many amphibian population declines remain enigmatic. For instance, in the eastern US – the center of global salamander diversity – Highton [8] described widespread declines in abundance in 180 of 205 populations of 38 *Plethodon* salamander species by the early 1980s, but he was unable to attribute a cause to most of the declines. Contemporary re-surveys of 72 of Highton's populations in Great Smoky Mountain National Park identified reduced occupancy and reduced abundance in 28 populations of six *Plethodon* taxa, yet *Bd* was not detected in any of the 485 *Plethodon* tested [9].

In this study, we tested the hypothesis that the disease chytridiomycosis, caused by *Bd* infection, was the driver of Appalachian *Plethodon* salamander declines reported in the 1980s. While the Great Smoky Mountain NP study [9] covered a wide range of elevations, it was restricted to contemporary field surveys of 35 sites in the Southern Appalachians. If *Bd* were responsible for the population declines in Appalachian *Plethodon*, we expected to detect an increase in prevalence of *Bd* in museum specimens collected prior to the reported declines in the 1970s as reported for Central American plethodontids [7]. To test this hypothesis we extended our surveys for *Bd* in both time and space. We sampled museum specimens collected by Highton and colleagues from 1957–1987 at five sites [7] and conducted field resurveys of 79 historic sites in 2011. We tested a subset of these samples for the newly describe salamander chytrid, *Batrachochytrium salamandivorans* [10] and for ranavirus [11].

## Methods

### Field Sampling

We worked with staff at the National Museum of Natural History to determine which historic collecting sites had the most extensive records to determine contemporary resurvey sites. From June–October 2011 we sampled all species of salamanders at 79 historic collecting sites originally visited by R. Highton and colleagues. At all sites we captured salamanders from 3, 50×3 m diurnal cover object plots where we turned over every log, rock or branch and captured all salamanders encountered. At some sites we also captured salamanders from 2, 50 m nocturnal visual encounter surveys where we captured every salamander seen along the transect. We identified each capture to species and sex, and measured snout-vent-length (SVL), mass, and body temperature. We swabbed each salamander 5 times on each limb and on the venter, and stored the swab in a 2 ml tubes with 70% ethanol. We used a new bag for each animal and changed gloves between captures to minimize the possibility of cross contamination, which also eliminated the need for disinfecting calipers. We accessed sites along well-traveled roads and hiking trails that were relatively dry, minimizing the need for disinfection.

To minimize cost of molecular analyses, we planned to establish *Bd* prevalence in the field by first testing all samples of the *P. cinereus* and *P. glutinosus* species groups. We focused on these groups because of their broad ranges, abundance in museum collections, and because we had noticed species-specific differences in the degree of decline in these two groups (unpublished data). We minimized sampling from the Great Smoky Mountain NP given our prior *Bd* surveys in this region [9]. Depending on the results of this first pass we planned additional sampling of other species and sites. To determine sampling effort necessary to detect *Bd*, we calculated the probability of detecting a false negative based on 5% infection prevalence, [4], [7] and we attempted to sample sufficient numbers per site or species to reach that level.

**Figure 1:**
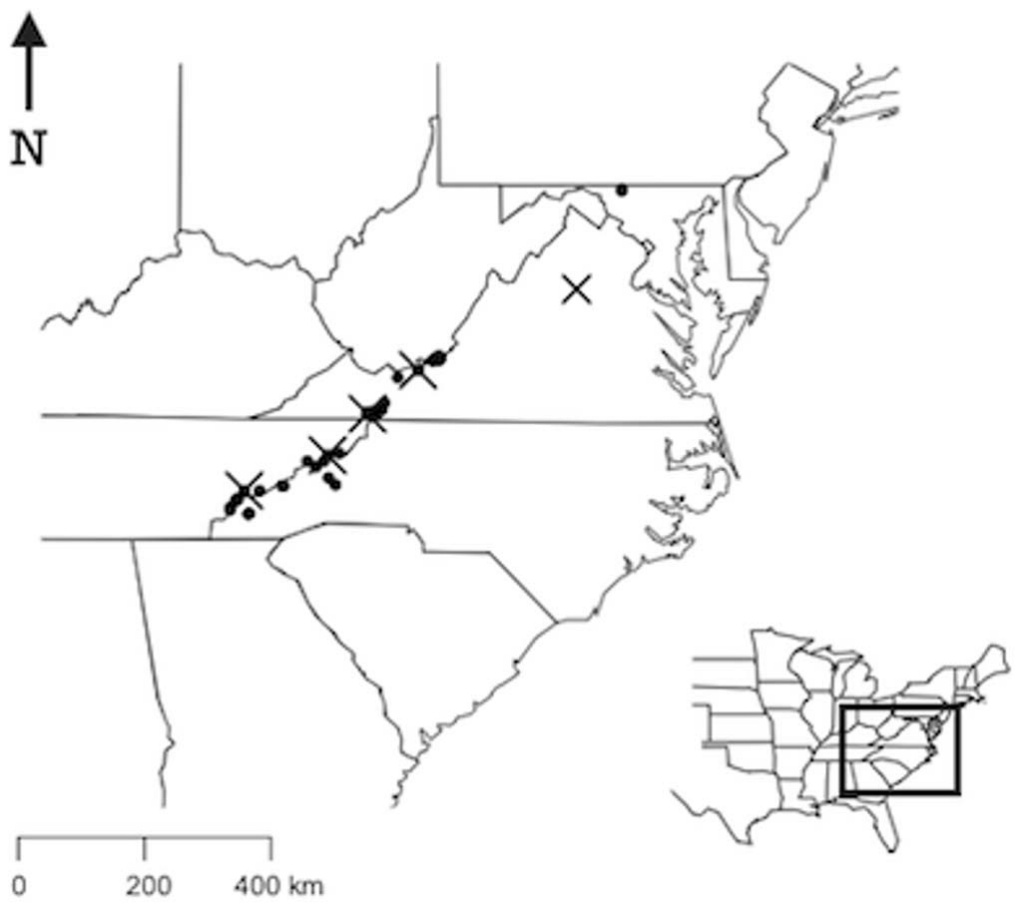
We sampled historic specimens collected at five sites (×), and live salamanders from 48 sites (•). Sampling sites spanned a geographical range of 767–1,687 m in the Appalachian Mountains. An inset of the US is provided to show the geographic extent.

In total, we tested samples from 48 of the 79 sites. The 48 sites were selected because *Plethodon* species still occurred there and because most of these sites had not already been sampled in 2009 [9]. The 48 sites spanned a geographical range of 767 km and an elevational range of 401–1,687 m (Figure 1) and were located within 5 protected areas across four states: Great Smoky Mountains National Park, TN and NC (8 sites), Nantahala National Forest, NC (4 sites), Pisgah National Forest, NC (9 sites), George Washington and Thomas Jefferson National Forests, VA (25 sites) and Catoctin Mountain Park, MD (2 sites).

### Museum Sampling

We worked with staff at the National Museum of Natural History to determine which specimens were available from each site to search for *Bd* in historic samples (Table S2). We selected five sites that had at least 100 *Plethodon* museum specimens collected prior to and during population declines reported in the 1980s [8]. Four of these sites were visited during field surveys in 2011: Dismal Creek, VA, Rust – Iron Mountain, VA, Indian Grave Gap, NC, and White Oak Sinks, TN. The fifth site, Hawksbill Mountain, VA had extensive museum holdings, but we were unable to sample in the field because of the presence of an endangered species (*P. shenandoah*).

To minimize cost of molecular analyses, we developed a plan for testing samples that would allow us to identify the location of the oldest *Bd*+ sample from museum holdings and determine any geographic spread by sampling out in time and space from that location. We started by sampling specimens of the *P. cinereus* and *P.glutinosus* species groups collected at Hawksbill Mountain (1957–1979, n = 365) and Indian Grave Gap, VA (1970–1987, n = 339), because these sites had the most extensive holdings, some of the oldest collections and together covered most of our spatial and temporal focus. We expected to find *Bd*+ animals prior to population declines in the 1980s and began by sampling roughly 60 specimens per species group per decade. Depending on what we found at these two sites, we planned similar sampling effort for specimens collected during the 1970s at the remaining three sites (Dismal Creek, VA: 1969–1974, n = 96, Rust – Iron Mountain, VA: 1972–1987, n = 61, and White Oak Sinks, TN: 1971–1974, n = 31), to establish the date and location of the oldest record and any potential geographic spread. To determine sampling effort necessary to detect *Bd*, we calculated the probability of detecting a false negative based on 5% infection prevalence [4], [7], and we ended sampling when we had sufficient numbers per site or species to reach that level.

We followed established methods [7], [12] to collect swabs from preserved specimens from the two species groups (Table S1). To decrease chances of contamination from errant pieces of skin or zoospores floating in the jar, we rinsed each salamander with 70% ethanol before swabbing, and held each specimen in a new plastic bag to minimize cross contamination from handling. We also collected swabs of the alcohol in each preservation jar to determine if that might be a source of contamination.

### DNA Extraction and PCR

We extracted DNA from the 2011 field swabs following the Qiagen BioSprint manual for Tissue Extraction using a three-hour incubation in lysis buffer. Prior to extraction, we removed residual ethanol from the swabs with a speed vacuum. For museum specimen swab samples, we performed DNA extractions in an ancient DNA lab at the Center for Conservation and Evolutionary Genetics. Ancient DNA labs are free from modern DNA, which can contaminate samples [13]. We extracted DNA from museum swabs following the Qiagen Blood and Tissue Kit 96 manual for Tissue Extraction using an overnight incubation in lysis buffer and a final elution volume of 80 ul. To verify that our DNA extraction methods could extract *Bd* DNA, we included known *Bd* positive control swabs from live and preserved frogs at the beginning and end of extracting the 2011 samples and the museum samples, respectively.

We used standard methods to quantify pathogen load using qPCR [7], [14] with minor modifications as follows. We used iTaq supermix with Rox (Bio-Rad) and followed their qPCR reaction protocol. We ran all contemporary DNA samples individually, and we re-ran positive samples in triplicate to verify and quantify *Bd* load in zoospore genomic equivalents (ZGEs) using standards of 100, 10, 1, 0.1 ZGEs developed from Puerto Rican isolate, JEL 427. Because DNA degrades over time, we are unable to estimate ZGEs of museum samples so we ran these samples in duplicate to verify *Bd* presence. *Bd* positive and negative PCR controls (water) were used in each qPCR run.

To verify that our protocol successfully extracted and amplified ancient DNA, in the potential absence of amplification of *Bd*, we developed primers to amplify *Plethodon* DNA (GenBank: JF731309.1, Pleth1F 5′ TCGGACGAGGAATTTATTATGG 3′ and Pleth1R 5′ GACATATCCTATGAAAGCTGTTGC 3′). We tested 36 *P. cinereus* and 31 *P. cylindraceus* DNA extracts using SybrGreen qPCR following the iTaq SybrGreen Supermix with Rox protocol (Bio-Rad).

We sequenced 21 putative *Bd* positive samples (four 2011 samples, 17 museum samples) using conventional PCR to amplify *Bd* DNA using the ITS and 5.8S primers [14]. We then cleaned amplified PCR products with ExoSAP-IT (United States Biochemical), and sequenced the cleaned amplicons using the BigDye Terminator Cycle Sequencing Kit (Applied Biosystems, Inc). The sequenced products were column filtered, dried down, rehydrated with 10 ul of HPLC purified formamide, and then analyzed on an Applied Biosystems 3130xl DNA Analyzer. Individual sequences were assembled and edited in Sequencher 5.1.

To test for the two other known amphibian pathogens we followed published PCR methods for *Bs* [10] and qPCR methods for ranavirus [15]. We tested a subset of 94 field samples from the two species groups (*P. cinereus*  = 21, *P. welleri*  = 5, *P. richmondi*  = 10, *P. cylindraceus*  = 19, *P. glutinosus*  = 31, *P. yonahlosee*  = 8) sampled across 26 sites to maximize geographic coverage.

### Statistics and Mapping

We calculated 95% confidence intervals (CIs) for our prevalence estimates for *Bd*, *Bs* and ranavirus. We used the package ‘Hmisc’ [16] in R [17]. We used the number of positives, the total number of individuals sampled, alpha  = 0.05 (Type I error) and the Wilson score interval for the CI estimates [18].

We used the package ‘maps’ [19] in R to geographically display the sites we sampled (Figure 1).

### Ethics Statement

We had permits from state and federal agencies for handling and swabbing live amphibians – Maryland (DNR Permit No. 50269), Virginia (VADGIF Permit No. 042151), North Carolina (Wildlife Resources Commission Permit No. 11-SC00547), Tennessee (Wildlife Resources Agency Permit No. 3636) and Great Smoky Mountains NP (Permit No. GRSM-2011-SCI-0059). We had permission from the National Museum of Natural History for sampling museum specimens. We received approval for the research from the University of Maryland Institutional Animal Care and Use Committee (R–11–11).

## Results

We surveyed 79 sites in 2011 and collected 1,436 swabs from 26 plethodontid salamander species representing *Plethodon*, *Eurycea* and *Desmognathus* genera. We tested 606 of those swabs for *Bd* from 11 species of our focal genus *Plethodon* (Table S1). Overall prevalence for *Plethodon* from 2011 field samples was 0.7% (95% CI: 0.3–1.7% prevalence; Table 1, Table 2). There were four *Bd*+ samples that all had low infection intensities (average zoospore genomic equivalents  = 7.9, range 2.3–20.4), and were all *P. cinereus* sampled from the same site in Catoctin Mountain Park, MD in October 2011 (Table 2). Three of the positive samples exactly matched *Bd* sequences in GenBank (accession# AY598034). The fourth sample did not produce a good quality sequence, likely because of low infection intensity, but amplified four times so we considered it a true positive following Hyatt *et al.,* [20]. Because of low number of positives we did not test the other taxa to reduce costs.

**Table 1:** Results of *Bd* testing for 12 species of *Plethodon* species.

	1957–1987 (Museum)		2011 (Field)	
Species	No. sampled	Prevalence (*95% CI*)	No. sampled	Prevalence (*95% CI*)
*P. welleri*	–	–	12	0 (*0*–*24*)
*P. cinereus*	561	0 (*0*–*0.7*)	396	1 (*0.3*–*2.5*)
*P. richmondi*	31	0 (*0*–*11*)	21	0 (*0*–*15*)
*P. serratus*	3	0 (*0*–*56*)	11	0 (*0*–*26*)
*P. shenandoah*	51	0 (*0*–*7*)	–	–
*P. glutinosus*	71	0 (*0*–*5.1*)	82	0 (*0*–*4.5*)
*P. aureoleus*	–	–	2	0 (*0*–*66*)
*P. cylindraceus*	175	0 (*0*–*2.4*)	38	0 (*0*–*9.4*)
*P. teyahalee*	–	–	20	0 (*0*–*16*)
*P. cheoah*	–	–	3	0 (*0*–*56*)
*P. montanus*	–	–	6	0 (*0*–*39*)
*P. yonahlosee*	–	–	15	0 (*0*–*20*)
**TOTAL**	**892**	**0 (** ***0–0.4*** **)**	**606**	**0.7 (** ***0.3–1.7*** **)**

Four present-day P. cinereus tested positive for Bd.

**Table 2:** Results of *Bd* testing for six protected areas in the Appalachian Mountains.

	1957–1987 (Museum)			2011 (Field)		
Protected Area	No. sites	No. sampled	Prevalence (*95% CI*)	No. sites	No. sampled	Prevalence (*95% CI*)
Catoctin Mountain Park, MD	–	–	–	2	54	7.4 (*2.9*–*18*)
Shenandoah National Park, VA	1	365	0 (*0*–*1*)	–	–	–
Washington and Jefferson National Forest, VA	2	157	0 (*0–2.4*)	25	456	0 (*0–0.8*)
Pisgah National Forest, NC	1	339	0 (*0–1.1*)	9	54	0 (*0–6.6*)
Nantahala National Forest, NC	–	–	–	4	8	0 (*0–32*)
Great Smoky Mountains National Park, NC/TN	1	31	0 (*0–11*)	8	34	0 (*0–10*)
**TOTAL**	**5**	**892**	**0 (** ***0–0.4*** **)**	**48**	**606**	**0.7 (** ***0.3–1.7*** **)**

We collected swabs from 892 museum specimens of six *Plethodon* species from 5 sites (Table S1). All museum specimens were negative for *Bd* (95% CI: 0–0.4% prevalence; Table 1, Table 2). Because we did not detect any positives at Hawksbill Mountain and Indian Grave Gap, VA we limited the number of samples at the three geographically intermediate sites to reduce cost. We tested swabs of the alcohol from 27 preservation jars for *Bd*, and all were negative.

We detected non-specific amplification in 23 of the samples after qPCR cycle 40, but we ruled these out as *Bd* when they did not re-amplify and the product sequences did not match expected *Bd* sequences. Sequences from nine putative *Bd* positive museum samples produced a 42-bp consensus sequence, excluding primers, which did not align with that of *Bd* or with any known sequence in GenBank. We were unable to deposit the consensus sequence in GenBank due to their requirements on minimum base pair size; it is provided here: 5′AACTAGTTATAGATGCCATGTATTGAGAATTGTGTTGCCGAA 3′. DNA extracted from the museum specimen swabs was of good quality as indicated by successfully amplification of salamander DNA from all 67 samples tested.

All of the 94 samples collected during 2011 field surveys from six *Plethodon* species were negative for both *Bs* and ranavirus, with prevalence estimates for each to be 0–4% using a 95% CI.

## Discussion

We found no evidence that chytridiomycosis, caused by *Bd* infection, was associated with historic declines of Appalachian *Plethodon* salamanders. Sample sizes were large enough to indicate with 95% confidence that the prevalence of *Bd* infection was below 2% for both our historic and contemporary samples. If we combine our dataset with all other published studies that have tested *Plethodon* spp. for *Bd* (Table 3), only 19 *Plethodon* salamanders out of 2,728 have tested positive for *Bd* (95% CI: 0.4–1.1% prevalence).

**Table 3:** Compilation of previous studies that have tested *Plethodon* salamanders for *Bd* infection in the eastern US.

Species	Location	N	N *Bd*+
*P. welleri*	North Carolina^[27]^	4	0
*P. ventralis*	Tennessee^[9]^	56	0
*P. cinereus*	Conneticut^[26]^, Virginia^[29], [31], [45]^	296	8
*P. richmondi*	North Carolina^[27]^	6	0
*P. serratus*	North Carolina^[23], [25]^, Tennessee^[9], [25]^	32	0
*P. glutinosus*	North Carolina^[27]^, Tennessee^[9]^	46	1
*P. cylindraceus*	Virginia^[45]^	19	0
*P. teyahalee*	North Carolina^[25]^, Tennessee^[9], [25]^	25	0
*P. yonahlossee*	North Carolina^[27]^	40	1
*P. jordani* and hybrids	North Carolina^[25]^, Tennessee^[9], [25]^	507	0
*P. metcalfi*	North Carolina^[23], [25], [27]^, Tennessee^[9], [25]^	104	0
*P. shermani*	North Carolina^[46]^	95	5
	**TOTAL:**	**1230**	**15**

We find it unusual that these Appalachian salamanders exhibit such low incidence of *Bd* infection for several reasons. First, *Bd* has been present in the Appalachians at least since the 1970s [21] and most surveys for *Bd* across the eastern US have produced prevalence estimates of 10–40% (e.g., [22]–[26]). Second, in laboratory infection trials *Plethodon* salamanders are susceptible to *Bd* [27], [28] and some die from *Bd* infection [29]. Third, the environment in which *Plethodon* salamanders live is suitable for *Bd* [9]. Finally, populations of terrestrial plethodontid salamanders from Central America have declined due to chytridiomycosis historically [7] and currently [3], [30].

We explain this low incidence of *Bd* with the following possibilities: 1) infected salamanders are not detected, 2) the Appalachians harbor a unique strain of chytrid fungus that does not amplify using these primers, and/or 3) *Plethodon* host traits limit *Bd* infection. We discuss each of these below as well as other potential causes of what remains enigmatic Appalachian *Plethodon* declines.

*Lack of detection*: If infected salamanders were present in the region, lack of detection might be explained if infected animals occupied unsampled habitats such as underground. Experimental studies have shown that when individuals of *P. cinereus, P. montanus,* and *P. glutinosus* salamanders are infected with high *Bd* levels >1×10^6^ zoospores) most ndividuals sustained an infection or xperienced ortality 5 to 43 days post exposure 27–29,31,32]. Given our extensive spatial and temporal sampling efforts, in addition to sampling by others (Table 3), we consider it likely that some *Bd* infected salamanders would have been detected, during the time course of infection, before they moved underground.

It is also possible that our lab procedures, although highly sensitive, missed some light *Bd* infections (e.g., [33]), especially in museum specimens where DNA degrades over time and is damaged in formalin. However, it is unlikely that we missed heavy *Bd* infections if they were present because previous studies [7] reported 78.1% accuracy detecting *Bd* infections of >300 ZGEs using duplicate qPCR runs from 40 year old museum specimens of Central American plethodontids.

*Pathogen strain*: If the eastern US had an endemic strain of chytrid (*e.g.,* [34], [35]), especially one that did not amplify with these primers, then this might explain differences in response of plethodontid salamanders in Central America [7] to that of eastern plethodontids [8], [9]. We have good evidence that *Bd* recently moved into Central America [3] and caused declines in plethodontid populations from Mexico through Panama [7], [30], but we have no evidence of these types of epidemics from the northeastern US. A long history of coexistence would likely be associated with low virulence of the pathogen [36], which may result in low infectivity. Few strains of *Bd* from the eastern and midwestern US have been cultured, leaving the possibility of an undescribed endemic strain in the region of our surveys.

*Host Traits*: Host defenses such as production of antimicrobial peptides [37], antibiosis by resident skin bacteria [31], [32], or genetic defenses [38] may contribute to low *Bd* infection prevalence. The only evidence for such host defenses in Appalachian populations is from *P. cinereus* in which the presence of certain antifungal bacteria can reduce *Bd* infections in the lab [29] likely through the production of toxic metabolites [39]. However, there has been no systematic survey of the geographic or taxonomic distribution of these innate and adaptive immune responses.

*Alternative causes*: If *Bd* did not cause these declines, then what other factors might cause such synchronous population declines in so many species and populations across the eastern US [8]? Previous studies from our lab [9], [40] have provided evidence that over-collecting, deforestation, and other anthropogenic factors were not involved and suggested that both disease and climate change are potential causes of these population declines [41].

A novel pathogen may be responsible for these *Plethodon* population declines. While our samples for *Batrachochytrium salamandrivorans* (*Bs*) and ranavirus were all negative, they were limited in number and only field samples were tested, leaving open the possibility that they were more prevalent historically. *Bs* is a recently discovered *Batrachochytrium* species associated with mortality in the fire salamander *Salamandra salamandra* in the Netherlands [10] that to date, has not been detected in the US. Ranavirus is widely distributed in the eastern US and has been associated with mass mortalities of aquatic-breeding amphibians [11]. Ranavirus has been detected at 80%, in salamander Great Smoky Mountains NP salamanders, primarily in aquatic species of *Desmognathus* [42]. Future studies should examine historical samples for the presence of *Bs* and ranavirus, and test response of *Plethodon* species to both pathogens in experiments.

Additionally, other as yet identified pathogens might be involved in the population declines. For example, the mysterious 42-bp consensus sequence we generated using *Bd*- specific primers from nine museum specimens may represent cryptic diversity of *Bd* in the eastern US, or it may simply be a spurious result from performing PCR on ancient DNA; we are actively investigating this.

Climate change may also be responsible for the observed *Plethodon* population declines. Recent work [40] presented evidence collected from these same surveys that populations of several *Plethodon* species are experiencing reductions in body size, with greater reduction in areas that have experienced warmer and drier conditions. This reduced body size may be related to the reduced abundance and occupancy in *Plethodon* populations [8], [9].

## Summary and Conclusion

Our extensive sampling across 6 decades, 767 km, 12 *Plethodon* taxa and 1400+ individuals was sufficient to have detected *Bd* if it were present in Appalachian *Plethodon* salamanders, at least as we currently understand *Bd* disease dynamics in wild populations (*e.g.,* [4], [43]). Therefore, we conclude that it is not a recent introduction of *Bd* that is responsible for the Appalachian *Plethodon* salamander declines. We also conclude that it is unlikely *Bs* or ranavirus given the lack of detection of these pathogens in 2011 field samples. Yet, widespread declines in amphibian populations are occurring across the United States [44] and globally [1], [2]. The need to identify the cause and timing of *Plethodon* salamander declines as well as enigmatic amphibian declines in the US and globally is critical for prioritizing conservation efforts.

## Supporting Information

Table S1 **Site and species summary of salamanders tested for** ***Bd*** **.** Number in parentheses indicate samples tested for both *Bs* and ranavirus.
(PDF)

Table S2 **National Museum of Natural History (USNM) specimen data for salamanders tested for** ***Bd*** **.**
(PDF)
