# Supplementary material for: Unexpected Rarity of the Pathogen Batrachochytrium dendrobatidis in Appalachian Plethodon Salamanders: 1957–2011
Source: PLoS One. 2014 Aug 1;9(8):e103728. doi: 10.1371/journal.pone.0103728 (PMC4118919; doi:10.1371/journal.pone.0103728)
Supplement: Table S1 — Site and species summary of salamanders tested for Bd . Number in parentheses indicate samples tested for both Bs and ranavirus. (PDF) [file pone.0103728.s001.pdf]

Site	Latitude	Longitude	Elevation (m)	State	Species	No. analyzed field	No. analyzed museum
Naked Ground (NG)	35.3811	-83.9842	1483.72	Tennessee	*P. aureoleus*	2	–
Sassafras Gap (SG)	35.3314	-83.6672	1336.86	North Carolina	*P. cheoah*	2	–
Bartram Trail A (BTA)	35.3242	-83.6847	1493.77	North Carolina	*P. cheoah*	1	–
Manahan Road (MNR)	39.6619	-77.4769	438.8	Maryland	*P. cinereus*	7	–
Foxville Deerfield Road (FDR)	39.6539	-77.4875	401.48	Maryland	*P. cinereus*	41	–
Hawksbill (HB)	38.5563	-78.3911	1076.86	Virginia	*P. cinereus*	–	315
White Rocks Camp Ground (WRCG)	37.4286	-80.4964	917.62	Virginia	*P. cinereus*	23	–
Mountain Lake Road A (MLA)	37.4228	-80.5047	1026.49	Virginia	*P. cinereus*	28	–
War Spur Trail Head (WST)	37.3906	-80.5086	1129.97	Virginia	*P. cinereus*	20	–
Moutain Lake Road B (MLB)	37.4208	-80.5097	1074.89	Virginia	*P. cinereus*	13	–
Appalachian Trail/Wind Rock (ATW)	37.4161	-80.5097	1247.46	Virginia	*P. cinereus*	25	–
War Spur A (WSA)	37.3883	-80.5108	1132.46	Virginia	*P. cinereus*	13	–
Potts Mountain Trail (PM)	37.415	-80.515	1256.55	Virginia	*P. cinereus*	13	–
Mountain Lake Road C (MLC)	37.4156	-80.5194	1181.97	Virginia	*P. cinereus*	13	–
Wind Rock (WR)	37.4122	-80.5228	1210.49	Virginia	*P. cinereus*	26 (1)	–
Appalachian Trail to Butt Mtn (ATBM)	37.4036	-80.5497	1141.78	Virginia	*P. cinereus*	20	–
Butt Mountain C (BMC)	37.3936	-80.5922	1164.45	Virginia	*P. cinereus*	10	–
Butt Mountain A (BMA)	37.3836	-80.6075	1157.48	Virginia	*P. cinereus*	26	–
Butt Mountain B (BMB)	37.3706	-80.6125	1254.46	Virginia	*P. cinereus*	11	–
Dismal Creek Rd. (DCR)	37.2489	-80.8633	1142.53	Virginia	*P. cinereus*	3	52
Round Mtn (RM)	37.1494	-81.2003	1140.26	Virginia	*P. cinereus*	6	–
Hurricane X Iron Mtn Trails (HIM)	36.7144	-81.4728	1052.2	Virginia	*P. cinereus*	2 (1)	–
Deep Gap Spring (DGS)	36.6578	-81.5569	1492.41	Virginia	*P. cinereus*	15 (1)	–
White Top Mountain Smyth Co. (WTMG)	36.6567	-81.5889	1217.12	Virginia	*P. cinereus*	3	–
White Top Mountain Road (WTMR)	36.6306	-81.5894	1427.69	Virginia	*P. cinereus*	10 (3)	–
White Top Mountain (WTM)	36.6389	-81.6069	1676.12	Virginia	*P. cinereus*	6 (3)	–
Iron Mtn. Rhododendron Stream (IMRS)	36.6994	-81.6239	1087.91	Virginia	*P. cinereus*	3 (2)	–
Lum/Iron Mountain (LIM)	36.6953	-81.6344	1172.59	Virginia	*P. cinereus*	9	–
Rust/Iron Mountain (RIM)	36.6653	-81.7025	1067.6	Virginia	*P. cinereus*	24 (7)	13
Rush trail and Sawmill Pond (RSM)	36.6747	-81.7042	900.06	Virginia	*P. cinereus*	2	–
Saw Mill Ridge (SMR)	36.6844	-81.7067	879.97	Virginia	*P. cinereus*	1	–
Feather Camp Ridge (FCR)	36.665	-81.7175	1147.89	Virginia	*P. cinereus*	5 (2)	–
Hughes Gap (HG)	36.1372	-82.1717	1104.97	North Carolina	*P. cinereus*	8	–
Indian Grave Gap (IGG)	36.11	-82.3611	1032.98	Tennessee	*P. cinereus*	2	181
Spivey Gap (SPG)	36.0328	-82.4258	996.15	Tennessee	*P. cinereus*	2	–
Sam's Gap Appalachian Trail (SGAT)	35.9542	-82.5594	1189.86	Tennessee	*P. cinereus*	5	–
White Cliff (WC)	36.0319	-82.7028	1343.73	Tennessee	*P. cinereus*	1	–
Hurricane X Iron Mtn Trails (HIM)	36.7144	-81.4728	1052.2	Virginia	*P. cylindraceus*	2	–
Rust/Iron Mountain (RIM)	36.6653	-81.7025	1067.6	Virginia	*P. cylindraceus*	11 (6)	17
Rush trail and Sawmill Pond (RSM)	36.6747	-81.7042	900.06	Virginia	*P. cylindraceus*	1	–
Hughes Gap (HG)	36.1372	-82.1717	1104.97	North Carolina	*P. cylindraceus*	6 (6)	–
Indian Grave Gap (IGG)	36.11	-82.3611	1032.98	Tennessee	*P. cylindraceus*	2 (1)	158
Sam's Gap Appalachian Trail (SGAT)	35.9542	-82.5594	1189.86	Tennessee	*P. cylindraceus*	9 (5)	–
Sassafras Gap (SG)	35.3314	-83.6672	1336.86	North Carolina	*P. cylindraceus*	4	–
Bartram Trail B (BBT)	35.3222	-83.6864	1449.17	North Carolina	*P. cylindraceus*	2	–
Spivey Gap (SPG)	36.0328	-82.4258	996.15	Tennessee	*P. cylindraceus*	1 (1)	–
Foxville Deerfield Road (FDR)	39.6539	-77.4875	401.48	Maryland	*P. glutinosus*	8 (3)	–
White Rocks Camp Ground (WRCG)	37.4286	-80.4964	917.62	Virginia	*P. glutinosus*	8 (4)	–
Mountain Lake Road A (MLA)	37.4228	-80.5047	1026.49	Virginia	*P. glutinosus*	7 (3)	–
War Spur Trail Head (WST)	37.3906	-80.5086	1129.97	Virginia	*P. glutinosus*	2	–
Appalachian Trail/Wind Rock (ATW)	37.4161	-80.5097	1247.46	Virginia	*P. glutinosus*	2	–
War Spur A (WSA)	37.3883	-80.5108	1132.46	Virginia	*P. glutinosus*	1	–
Potts Mountain Trail (PM)	37.415	-80.515	1256.55	Virginia	*P. glutinosus*	2	–
Mountain Lake Road C (MLC)	37.4156	-80.5194	1181.97	Virginia	*P. glutinosus*	2 (2)	–
Wind Rock (WR)	37.4122	-80.5228	1210.49	Virginia	*P. glutinosus*	7 (4)	–
Butt Mountain C (BMC)	37.3936	-80.5922	1164.45	Virginia	*P. glutinosus*	3	–
Butt Mountain A (BMA)	37.3836	-80.6075	1157.48	Virginia	*P. glutinosus*	4	–
Butt Mountain B (BMB)	37.3706	-80.6125	1254.46	Virginia	*P. glutinosus*	6 (3)	–
Dismal Creek Rd. (DCR)	37.2489	-80.8633	1142.53	Virginia	*P. glutinosus*	–	43
Round Mtn (RM)	37.1494	-81.2003	1140.26	Virginia	*P. glutinosus*	6 (2)	–
Mt. Rogers National Recreation Area (NRA)	36.8119	-81.4208	991.02	Virginia	*P. glutinosus*	18 (10)	–
White Oak Sinks (WOS)	35.63889	-83.7478	550.88	Tennessee	*P. glutinosus*	6	28
Deep Gap Spring (DGS)	36.6578	-81.5569	1492.41	Virginia	*P. montanus*	3	–

White Top Mountain (WTM)	36.6389	-81.6069	1676.12	Virginia	*P. montanus*	1	–
Blue Ridge Pkwy (BRP)	35.71561	-82.2347	1366.63	North Carolina	*P. montanus*	1	–
White Cliff (WC)	36.0319	-82.7028	1343.73	Tennessee	*P. montanus*	1	–
Mt. Rogers National Recreation Area (NRA)	36.8119	-81.4208	991.02	Virginia	*P. richmondi*	3	–
Hurricane X Iron Mtn Trails (HIM)	36.7144	-81.4728	1052.2	Virginia	*P. richmondi*	1 (1)	–
White Top Mountain Smyth Co. (WTMG)	36.6567	-81.5889	1217.12	Virginia	*P. richmondi*	3	–
White Top Mountain Road (WTMR)	36.6306	-81.5894	1427.69	Virginia	*P. richmondi*	3	–
Lum/Iron Mountain (LIM)	36.6953	-81.6344	1172.59	Virginia	*P. richmondi*	4 (3)	–
Rust/Iron Mountain (RIM)	36.6653	-81.7025	1067.6	Virginia	*P. richmondi*	6 (5)	31
Rush trail and Sawmill Pond (RSM)	36.6747	-81.7042	900.06	Virginia	*P. richmondi*	1	–
Mt. Sterling A (MSA)	35.69972	-83.0981	1179.9	North Carolina	*P. serratus*	2	–
Mt. Sterling C (MSC)	35.70333	-83.11	1420.73	North Carolina	*P. serratus*	1	–
Chimney Picnic Area (CP)	35.63639	-83.4947	836.91	Tennessee	*P. serratus*	7	–
White Oak Sinks (WOS)	35.63889	-83.7478	550.88	Tennessee	*P. serratus*	–	3
Gregory Bald B (GBB)	35.52083	-83.8544	1396.17	Tennessee	*P. serratus*	1	–
Hawksbill (HB)	38.5563	-78.3911	1076.86	Virginia	*P. shenandoah*	–	51
Mt. Sterling A (MSA)	35.69972	-83.0981	1179.9	North Carolina	*P. teyahalee*	6	–
Mt. Sterling B (MSB)	35.70111	-83.1086	1383.02	North Carolina	*P. teyahalee*	6	–
Chimney Picnic Area (CP)	35.63639	-83.4947	836.91	Tennessee	*P. teyahalee*	2	–
Gregory Bald E (GBE)	35.51667	-83.8819	1432.06	Tennessee	*P. teyahalee*	2	–
Big Fat Gap (BFG)	35.4164	-83.9736	950.31	North Carolina	*P. teyahalee*	4	–
Deep Gap Spring (DGS)	36.6578	-81.5569	1492.41	Virginia	*P. welleri*	9 (5)	–
Indian Grave Gap (IGG)	36.11	-82.3611	1032.98	Tennessee	*P. welleri*	3	–
Rust/Iron Mountain (RIM)	36.6653	-81.7025	1067.6	Virginia	*P. yonahlosee*	4 (4)	–
Feather Camp Ridge (FCR)	36.665	-81.7175	1147.89	Virginia	*P. yonahlosee*	3	–
Big Butt Trail (BBT)	35.8083	-82.3481	1296.78	North Carolina	*P. yonahlosee*	2 (1)	–
Indian Grave Gap (IGG)	36.11	-82.3611	1032.98	Tennessee	*P. yonahlosee*	1	–
Sam's Gap Appalachian Trail (SGAT)	35.9542	-82.5594	1189.86	Tennessee	*P. yonahlosee*	5 (2)	–
					**TOTAL**	**606 (94)**	**892**
