# Supplementary material for: Unexpected Rarity of the Pathogen Batrachochytrium dendrobatidis in Appalachian Plethodon Salamanders: 1957–2011
Source: PLoS One. 2014 Aug 1;9(8):e103728. doi: 10.1371/journal.pone.0103728 (PMC4118919; doi:10.1371/journal.pone.0103728)
Supplement: Table S2 — National Museum of Natural History (USNM) specimen data for salamanders tested for Bd . (PDF) [file pone.0103728.s002.pdf]

USNM ID	Species	Collecting date	Site	Latitude	Longitude
431473	*P. cinereus*	28-Jul-69	DCR	37.2489	-80.8633
431474	*P. cinereus*	28-Jul-69	DCR	37.2489	-80.8633
431478	*P. cinereus*	28-Jul-69	DCR	37.2489	-80.8633
431517	*P. cinereus*	14-Aug-71	DCR	37.2489	-80.8633
431518	*P. cinereus*	14-Aug-71	DCR	37.2489	-80.8633
431519	*P. cinereus*	14-Aug-71	DCR	37.2489	-80.8633
431516	*P. cinereus*	14-Aug-71	DCR	37.2489	-80.8633
431576	*P. cinereus*	27-Jul-72	DCR	37.2489	-80.8633
431577	*P. cinereus*	27-Jul-72	DCR	37.2489	-80.8633
431578	*P. cinereus*	27-Jul-72	DCR	37.2489	-80.8633
431579	*P. cinereus*	27-Jul-72	DCR	37.2489	-80.8633
431580	*P. cinereus*	27-Jul-72	DCR	37.2489	-80.8633
431581	*P. cinereus*	27-Jul-72	DCR	37.2489	-80.8633
431584	*P. cinereus*	27-Jul-72	DCR	37.2489	-80.8633
431585	*P. cinereus*	27-Jul-72	DCR	37.2489	-80.8633
431586	*P. cinereus*	27-Jul-72	DCR	37.2489	-80.8633
431587	*P. cinereus*	27-Jul-72	DCR	37.2489	-80.8633
431618	*P. cinereus*	22-Jul-73	DCR	37.2489	-80.8633
431620	*P. cinereus*	22-Jul-73	DCR	37.2489	-80.8633
431663	*P. cinereus*	28-Sep-73	DCR	37.2489	-80.8633
431664	*P. cinereus*	28-Sep-73	DCR	37.2489	-80.8633
431665	*P. cinereus*	28-Sep-73	DCR	37.2489	-80.8633
431666	*P. cinereus*	28-Sep-73	DCR	37.2489	-80.8633
431667	*P. cinereus*	28-Sep-73	DCR	37.2489	-80.8633
431668	*P. cinereus*	28-Sep-73	DCR	37.2489	-80.8633
431669	*P. cinereus*	28-Sep-73	DCR	37.2489	-80.8633
431670	*P. cinereus*	28-Sep-73	DCR	37.2489	-80.8633
431671	*P. cinereus*	28-Sep-73	DCR	37.2489	-80.8633
431672	*P. cinereus*	28-Sep-73	DCR	37.2489	-80.8633
431673	*P. cinereus*	28-Sep-73	DCR	37.2489	-80.8633
431674	*P. cinereus*	28-Sep-73	DCR	37.2489	-80.8633
471995	*P. cinereus*	13-Oct-73	DCR	37.2489	-80.8633
471996	*P. cinereus*	13-Oct-73	DCR	37.2489	-80.8633
471997	*P. cinereus*	13-Oct-73	DCR	37.2489	-80.8633
471998	*P. cinereus*	13-Oct-73	DCR	37.2489	-80.8633
471999	*P. cinereus*	13-Oct-73	DCR	37.2489	-80.8633
472001	*P. cinereus*	13-Oct-73	DCR	37.2489	-80.8633
472002	*P. cinereus*	13-Oct-73	DCR	37.2489	-80.8633
472003	*P. cinereus*	13-Oct-73	DCR	37.2489	-80.8633
472004	*P. cinereus*	13-Oct-73	DCR	37.2489	-80.8633
472005	*P. cinereus*	13-Oct-73	DCR	37.2489	-80.8633
431712	*P. cinereus*	24-Jun-74	DCR	37.2489	-80.8633
431713	*P. cinereus*	24-Jun-74	DCR	37.2489	-80.8633
431715	*P. cinereus*	24-Jun-74	DCR	37.2489	-80.8633
431716	*P. cinereus*	24-Jun-74	DCR	37.2489	-80.8633
431718	*P. cinereus*	24-Jun-74	DCR	37.2489	-80.8633
431719	*P. cinereus*	24-Jun-74	DCR	37.2489	-80.8633
431720	*P. cinereus*	24-Jun-74	DCR	37.2489	-80.8633

431721	*P. cinereus*	24-Jun-74	DCR	37.2489	-80.8633
431724	*P. cinereus*	24-Jun-74	DCR	37.2489	-80.8633
431725	*P. cinereus*	24-Jun-74	DCR	37.2489	-80.8633
431727	*P. cinereus*	24-Jun-74	DCR	37.2489	-80.8633
431521	*P. glutinosus*	14-Aug-71	DCR	37.2489	-80.8633
431522	*P. glutinosus*	14-Aug-71	DCR	37.2489	-80.8633
431523	*P. glutinosus*	14-Aug-71	DCR	37.2489	-80.8633
431524	*P. glutinosus*	14-Aug-71	DCR	37.2489	-80.8633
431525	*P. glutinosus*	14-Aug-71	DCR	37.2489	-80.8633
431526	*P. glutinosus*	14-Aug-71	DCR	37.2489	-80.8633
431527	*P. glutinosus*	14-Aug-71	DCR	37.2489	-80.8633
431528	*P. glutinosus*	14-Aug-71	DCR	37.2489	-80.8633
431529	*P. glutinosus*	14-Aug-71	DCR	37.2489	-80.8633
431530	*P. glutinosus*	14-Aug-71	DCR	37.2489	-80.8633
431531	*P. glutinosus*	14-Aug-71	DCR	37.2489	-80.8633
431532	*P. glutinosus*	14-Aug-71	DCR	37.2489	-80.8633
431588	*P. glutinosus*	27-Jul-72	DCR	37.2489	-80.8633
431589	*P. glutinosus*	27-Jul-72	DCR	37.2489	-80.8633
431590	*P. glutinosus*	27-Jul-72	DCR	37.2489	-80.8633
431591	*P. glutinosus*	27-Jul-72	DCR	37.2489	-80.8633
431592	*P. glutinosus*	27-Jul-72	DCR	37.2489	-80.8633
431593	*P. glutinosus*	27-Jul-72	DCR	37.2489	-80.8633
431594	*P. glutinosus*	27-Jul-72	DCR	37.2489	-80.8633
431595	*P. glutinosus*	27-Jul-72	DCR	37.2489	-80.8633
431596	*P. glutinosus*	27-Jul-72	DCR	37.2489	-80.8633
431597	*P. glutinosus*	27-Jul-72	DCR	37.2489	-80.8633
431611	*P. glutinosus*	22-Jul-73	DCR	37.2489	-80.8633
431612	*P. glutinosus*	22-Jul-73	DCR	37.2489	-80.8633
431613	*P. glutinosus*	22-Jul-73	DCR	37.2489	-80.8633
431614	*P. glutinosus*	22-Jul-73	DCR	37.2489	-80.8633
431615	*P. glutinosus*	22-Jul-73	DCR	37.2489	-80.8633
431616	*P. glutinosus*	22-Jul-73	DCR	37.2489	-80.8633
431617	*P. glutinosus*	22-Jul-73	DCR	37.2489	-80.8633
431685	*P. glutinosus*	28-Sep-73	DCR	37.2489	-80.8633
431686	*P. glutinosus*	28-Sep-73	DCR	37.2489	-80.8633
431688	*P. glutinosus*	28-Sep-73	DCR	37.2489	-80.8633
431689	*P. glutinosus*	28-Sep-73	DCR	37.2489	-80.8633
431690	*P. glutinosus*	28-Sep-73	DCR	37.2489	-80.8633
431691	*P. glutinosus*	28-Sep-73	DCR	37.2489	-80.8633
432724	*P. glutinosus*	28-Sep-73	DCR	37.2489	-80.8633
432725	*P. glutinosus*	28-Sep-73	DCR	37.2489	-80.8633
432726	*P. glutinosus*	28-Sep-73	DCR	37.2489	-80.8633
432727	*P. glutinosus*	28-Sep-73	DCR	37.2489	-80.8633
432728	*P. glutinosus*	28-Sep-73	DCR	37.2489	-80.8633
431704	*P. glutinosus*	13-Oct-73	DCR	37.2489	-80.8633
431705	*P. glutinosus*	13-Oct-73	DCR	37.2489	-80.8633
431709	*P. glutinosus*	13-Oct-73	DCR	37.2489	-80.8633
415683	*P. cinereus*	13-Jul-57	HB	38.5563	-78.3911
415685	*P. cinereus*	13-Jul-57	HB	38.5563	-78.3911

415686	*P. cinereus*	13-Jul-57	HB	38.5563	-78.3911
415687	*P. cinereus*	13-Jul-57	HB	38.5563	-78.3911
415688	*P. cinereus*	13-Jul-57	HB	38.5563	-78.3911
415689	*P. cinereus*	13-Jul-57	HB	38.5563	-78.3911
415690	*P. cinereus*	13-Jul-57	HB	38.5563	-78.3911
415691	*P. cinereus*	13-Jul-57	HB	38.5563	-78.3911
415692	*P. cinereus*	13-Jul-57	HB	38.5563	-78.3911
415693	*P. cinereus*	13-Jul-57	HB	38.5563	-78.3911
415695	*P. cinereus*	13-Jul-57	HB	38.5563	-78.3911
415696	*P. cinereus*	13-Jul-57	HB	38.5563	-78.3911
415697	*P. cinereus*	13-Jul-57	HB	38.5563	-78.3911
415698	*P. cinereus*	13-Jul-57	HB	38.5563	-78.3911
415699	*P. cinereus*	13-Jul-57	HB	38.5563	-78.3911
415700	*P. cinereus*	13-Jul-57	HB	38.5563	-78.3911
415701	*P. cinereus*	13-Jul-57	HB	38.5563	-78.3911
415702	*P. cinereus*	13-Jul-57	HB	38.5563	-78.3911
415703	*P. cinereus*	13-Jul-57	HB	38.5563	-78.3911
415704	*P. cinereus*	13-Jul-57	HB	38.5563	-78.3911
415705	*P. cinereus*	13-Jul-57	HB	38.5563	-78.3911
415706	*P. cinereus*	13-Jul-57	HB	38.5563	-78.3911
415707	*P. cinereus*	13-Jul-57	HB	38.5563	-78.3911
415708	*P. cinereus*	13-Jul-57	HB	38.5563	-78.3911
415710	*P. cinereus*	13-Jul-57	HB	38.5563	-78.3911
415712	*P. cinereus*	13-Jul-57	HB	38.5563	-78.3911
415713	*P. cinereus*	13-Jul-57	HB	38.5563	-78.3911
415714	*P. cinereus*	13-Jul-57	HB	38.5563	-78.3911
415715	*P. cinereus*	13-Jul-57	HB	38.5563	-78.3911
415717	*P. cinereus*	13-Jul-57	HB	38.5563	-78.3911
415718	*P. cinereus*	13-Jul-57	HB	38.5563	-78.3911
415719	*P. cinereus*	13-Jul-57	HB	38.5563	-78.3911
415720	*P. cinereus*	13-Jul-57	HB	38.5563	-78.3911
415722	*P. cinereus*	13-Jul-57	HB	38.5563	-78.3911
415734	*P. cinereus*	5-Sep-58	HB	38.5563	-78.3911
415736	*P. cinereus*	5-Sep-58	HB	38.5563	-78.3911
415737	*P. cinereus*	5-Sep-58	HB	38.5563	-78.3911
415738	*P. cinereus*	5-Sep-58	HB	38.5563	-78.3911
415739	*P. cinereus*	5-Sep-58	HB	38.5563	-78.3911
415740	*P. cinereus*	5-Sep-58	HB	38.5563	-78.3911
415741	*P. cinereus*	5-Sep-58	HB	38.5563	-78.3911
415742	*P. cinereus*	5-Sep-58	HB	38.5563	-78.3911
415743	*P. cinereus*	5-Sep-58	HB	38.5563	-78.3911
415745	*P. cinereus*	5-Sep-58	HB	38.5563	-78.3911
415746	*P. cinereus*	5-Sep-58	HB	38.5563	-78.3911
415747	*P. cinereus*	5-Sep-58	HB	38.5563	-78.3911
415748	*P. cinereus*	5-Sep-58	HB	38.5563	-78.3911
415750	*P. cinereus*	5-Sep-58	HB	38.5563	-78.3911
415751	*P. cinereus*	5-Sep-58	HB	38.5563	-78.3911
415752	*P. cinereus*	5-Sep-58	HB	38.5563	-78.3911
415753	*P. cinereus*	5-Sep-58	HB	38.5563	-78.3911

415755	*P. cinereus*	5-Sep-58	HB	38.5563	-78.3911
415756	*P. cinereus*	5-Sep-58	HB	38.5563	-78.3911
415758	*P. cinereus*	5-Sep-58	HB	38.5563	-78.3911
415759	*P. cinereus*	5-Sep-58	HB	38.5563	-78.3911
415760	*P. cinereus*	5-Sep-58	HB	38.5563	-78.3911
415761	*P. cinereus*	5-Sep-58	HB	38.5563	-78.3911
415762	*P. cinereus*	5-Sep-58	HB	38.5563	-78.3911
415763	*P. cinereus*	5-Sep-58	HB	38.5563	-78.3911
415764	*P. cinereus*	5-Sep-58	HB	38.5563	-78.3911
415765	*P. cinereus*	5-Sep-58	HB	38.5563	-78.3911
415766	*P. cinereus*	5-Sep-58	HB	38.5563	-78.3911
415767	*P. cinereus*	5-Sep-58	HB	38.5563	-78.3911
415768	*P. cinereus*	5-Sep-58	HB	38.5563	-78.3911
415769	*P. cinereus*	5-Sep-58	HB	38.5563	-78.3911
415770	*P. cinereus*	5-Sep-58	HB	38.5563	-78.3911
415771	*P. cinereus*	5-Sep-58	HB	38.5563	-78.3911
415772	*P. cinereus*	5-Sep-58	HB	38.5563	-78.3911
415775	*P. cinereus*	5-Sep-58	HB	38.5563	-78.3911
415776	*P. cinereus*	5-Sep-58	HB	38.5563	-78.3911
415777	*P. cinereus*	5-Sep-58	HB	38.5563	-78.3911
415778	*P. cinereus*	5-Sep-58	HB	38.5563	-78.3911
415779	*P. cinereus*	5-Sep-58	HB	38.5563	-78.3911
415780	*P. cinereus*	5-Sep-58	HB	38.5563	-78.3911
415781	*P. cinereus*	5-Sep-58	HB	38.5563	-78.3911
415782	*P. cinereus*	5-Sep-58	HB	38.5563	-78.3911
415783	*P. cinereus*	5-Sep-58	HB	38.5563	-78.3911
415784	*P. cinereus*	5-Sep-58	HB	38.5563	-78.3911
415795	*P. cinereus*	5-Sep-58	HB	38.5563	-78.3911
415798	*P. cinereus*	15-Oct-64	HB	38.5563	-78.3911
415800	*P. cinereus*	15-Oct-64	HB	38.5563	-78.3911
415804	*P. cinereus*	15-Oct-64	HB	38.5563	-78.3911
415805	*P. cinereus*	15-Oct-64	HB	38.5563	-78.3911
415806	*P. cinereus*	15-Oct-64	HB	38.5563	-78.3911
415807	*P. cinereus*	15-Oct-64	HB	38.5563	-78.3911
415808	*P. cinereus*	15-Oct-64	HB	38.5563	-78.3911
415809	*P. cinereus*	15-Oct-64	HB	38.5563	-78.3911
415810	*P. cinereus*	15-Oct-64	HB	38.5563	-78.3911
415811	*P. cinereus*	15-Oct-64	HB	38.5563	-78.3911
415812	*P. cinereus*	15-Oct-64	HB	38.5563	-78.3911
415813	*P. cinereus*	15-Oct-64	HB	38.5563	-78.3911
415825	*P. cinereus*	15-Oct-64	HB	38.5563	-78.3911
415826	*P. cinereus*	15-Oct-64	HB	38.5563	-78.3911
415827	*P. cinereus*	15-Oct-64	HB	38.5563	-78.3911
415828	*P. cinereus*	15-Oct-64	HB	38.5563	-78.3911
415829	*P. cinereus*	15-Oct-64	HB	38.5563	-78.3911
415830	*P. cinereus*	15-Oct-64	HB	38.5563	-78.3911
415831	*P. cinereus*	15-Oct-64	HB	38.5563	-78.3911
415832	*P. cinereus*	15-Oct-64	HB	38.5563	-78.3911
415833	*P. cinereus*	15-Oct-64	HB	38.5563	-78.3911

415834	*P. cinereus*	15-Oct-64	HB	38.5563	-78.3911
415835	*P. cinereus*	15-Oct-64	HB	38.5563	-78.3911
415836	*P. cinereus*	15-Oct-64	HB	38.5563	-78.3911
415837	*P. cinereus*	9-Apr-65	HB	38.5563	-78.3911
415841	*P. cinereus*	9-Apr-65	HB	38.5563	-78.3911
415842	*P. cinereus*	9-Apr-65	HB	38.5563	-78.3911
415843	*P. cinereus*	9-Apr-65	HB	38.5563	-78.3911
415844	*P. cinereus*	9-Apr-65	HB	38.5563	-78.3911
415845	*P. cinereus*	9-Apr-65	HB	38.5563	-78.3911
415846	*P. cinereus*	9-Apr-65	HB	38.5563	-78.3911
415847	*P. cinereus*	9-Apr-65	HB	38.5563	-78.3911
415848	*P. cinereus*	9-Apr-65	HB	38.5563	-78.3911
415849	*P. cinereus*	9-Apr-65	HB	38.5563	-78.3911
415851	*P. cinereus*	9-Apr-65	HB	38.5563	-78.3911
415852	*P. cinereus*	9-Apr-65	HB	38.5563	-78.3911
415853	*P. cinereus*	9-Apr-65	HB	38.5563	-78.3911
415854	*P. cinereus*	9-Apr-65	HB	38.5563	-78.3911
415855	*P. cinereus*	9-Apr-65	HB	38.5563	-78.3911
415856	*P. cinereus*	9-Apr-65	HB	38.5563	-78.3911
415857	*P. cinereus*	9-Apr-65	HB	38.5563	-78.3911
415858	*P. cinereus*	9-Apr-65	HB	38.5563	-78.3911
415859	*P. cinereus*	9-Apr-65	HB	38.5563	-78.3911
415860	*P. cinereus*	9-Apr-65	HB	38.5563	-78.3911
415861	*P. cinereus*	9-Apr-65	HB	38.5563	-78.3911
415862	*P. cinereus*	9-Apr-65	HB	38.5563	-78.3911
415863	*P. cinereus*	9-Apr-65	HB	38.5563	-78.3911
415864	*P. cinereus*	9-Apr-65	HB	38.5563	-78.3911
415865	*P. cinereus*	9-Apr-65	HB	38.5563	-78.3911
415866	*P. cinereus*	9-Apr-65	HB	38.5563	-78.3911
415867	*P. cinereus*	9-Apr-65	HB	38.5563	-78.3911
415868	*P. cinereus*	9-Apr-65	HB	38.5563	-78.3911
415869	*P. cinereus*	9-Apr-65	HB	38.5563	-78.3911
415870	*P. cinereus*	9-Apr-65	HB	38.5563	-78.3911
415871	*P. cinereus*	9-Apr-65	HB	38.5563	-78.3911
415872	*P. cinereus*	9-Apr-65	HB	38.5563	-78.3911
415873	*P. cinereus*	9-May-65	HB	38.5563	-78.3911
415875	*P. cinereus*	9-May-65	HB	38.5563	-78.3911
415876	*P. cinereus*	9-May-65	HB	38.5563	-78.3911
415878	*P. cinereus*	9-May-65	HB	38.5563	-78.3911
415879	*P. cinereus*	9-May-65	HB	38.5563	-78.3911
415880	*P. cinereus*	9-May-65	HB	38.5563	-78.3911
415882	*P. cinereus*	9-May-65	HB	38.5563	-78.3911
415883	*P. cinereus*	9-May-65	HB	38.5563	-78.3911
415884	*P. cinereus*	9-May-65	HB	38.5563	-78.3911
415885	*P. cinereus*	9-May-65	HB	38.5563	-78.3911
415886	*P. cinereus*	9-May-65	HB	38.5563	-78.3911
415887	*P. cinereus*	9-May-65	HB	38.5563	-78.3911
415888	*P. cinereus*	9-May-65	HB	38.5563	-78.3911
415889	*P. cinereus*	9-May-65	HB	38.5563	-78.3911

415890	*P. cinereus*	9-May-65	HB	38.5563	-78.3911
415891	*P. cinereus*	9-May-65	HB	38.5563	-78.3911
415892	*P. cinereus*	9-May-65	HB	38.5563	-78.3911
415893	*P. cinereus*	9-May-65	HB	38.5563	-78.3911
415894	*P. cinereus*	9-May-65	HB	38.5563	-78.3911
415895	*P. cinereus*	9-May-65	HB	38.5563	-78.3911
415897	*P. cinereus*	9-May-65	HB	38.5563	-78.3911
415898	*P. cinereus*	9-May-65	HB	38.5563	-78.3911
415899	*P. cinereus*	9-May-65	HB	38.5563	-78.3911
415900	*P. cinereus*	9-May-65	HB	38.5563	-78.3911
415901	*P. cinereus*	9-May-65	HB	38.5563	-78.3911
415902	*P. cinereus*	9-May-65	HB	38.5563	-78.3911
415904	*P. cinereus*	9-May-65	HB	38.5563	-78.3911
415905	*P. cinereus*	9-May-65	HB	38.5563	-78.3911
415907	*P. cinereus*	9-May-65	HB	38.5563	-78.3911
415909	*P. cinereus*	9-May-65	HB	38.5563	-78.3911
415911	*P. cinereus*	9-May-65	HB	38.5563	-78.3911
415913	*P. cinereus*	9-May-65	HB	38.5563	-78.3911
415914	*P. cinereus*	9-May-65	HB	38.5563	-78.3911
415915	*P. cinereus*	9-May-65	HB	38.5563	-78.3911
415916	*P. cinereus*	9-May-65	HB	38.5563	-78.3911
415917	*P. cinereus*	9-May-65	HB	38.5563	-78.3911
415918	*P. cinereus*	9-May-65	HB	38.5563	-78.3911
415919	*P. cinereus*	9-May-65	HB	38.5563	-78.3911
415922	*P. cinereus*	9-May-65	HB	38.5563	-78.3911
415926	*P. cinereus*	9-May-65	HB	38.5563	-78.3911
415927	*P. cinereus*	9-May-65	HB	38.5563	-78.3911
415928	*P. cinereus*	9-May-65	HB	38.5563	-78.3911
415929	*P. cinereus*	9-May-65	HB	38.5563	-78.3911
415930	*P. cinereus*	9-May-65	HB	38.5563	-78.3911
415931	*P. cinereus*	9-May-65	HB	38.5563	-78.3911
415932	*P. cinereus*	9-May-65	HB	38.5563	-78.3911
415933	*P. cinereus*	9-May-65	HB	38.5563	-78.3911
415937	*P. cinereus*	9-May-65	HB	38.5563	-78.3911
415940	*P. cinereus*	9-May-65	HB	38.5563	-78.3911
486102	*P. cinereus*	15-May-71	HB	38.5563	-78.3911
486103	*P. cinereus*	15-May-71	HB	38.5563	-78.3911
486104	*P. cinereus*	15-May-71	HB	38.5563	-78.3911
486105	*P. cinereus*	15-May-71	HB	38.5563	-78.3911
486106	*P. cinereus*	15-May-71	HB	38.5563	-78.3911
486107	*P. cinereus*	15-May-71	HB	38.5563	-78.3911
486108	*P. cinereus*	15-May-71	HB	38.5563	-78.3911
486109	*P. cinereus*	15-May-71	HB	38.5563	-78.3911
486110	*P. cinereus*	15-May-71	HB	38.5563	-78.3911
486111	*P. cinereus*	15-May-71	HB	38.5563	-78.3911
486112	*P. cinereus*	15-May-71	HB	38.5563	-78.3911
486113	*P. cinereus*	15-May-71	HB	38.5563	-78.3911
486114	*P. cinereus*	15-May-71	HB	38.5563	-78.3911
486115	*P. cinereus*	15-May-71	HB	38.5563	-78.3911

486116	*P. cinereus*	15-May-71	HB	38.5563	-78.3911
486117	*P. cinereus*	15-May-71	HB	38.5563	-78.3911
486118	*P. cinereus*	15-May-71	HB	38.5563	-78.3911
486119	*P. cinereus*	15-May-71	HB	38.5563	-78.3911
486120	*P. cinereus*	15-May-71	HB	38.5563	-78.3911
486121	*P. cinereus*	15-May-71	HB	38.5563	-78.3911
486122	*P. cinereus*	15-May-71	HB	38.5563	-78.3911
486123	*P. cinereus*	15-May-71	HB	38.5563	-78.3911
485942	*P. cinereus*	16-May-71	HB	38.5563	-78.3911
485943	*P. cinereus*	16-May-71	HB	38.5563	-78.3911
485944	*P. cinereus*	16-May-71	HB	38.5563	-78.3911
485945	*P. cinereus*	16-May-71	HB	38.5563	-78.3911
485946	*P. cinereus*	16-May-71	HB	38.5563	-78.3911
485947	*P. cinereus*	16-May-71	HB	38.5563	-78.3911
485948	*P. cinereus*	16-May-71	HB	38.5563	-78.3911
485949	*P. cinereus*	16-May-71	HB	38.5563	-78.3911
485950	*P. cinereus*	16-May-71	HB	38.5563	-78.3911
485951	*P. cinereus*	16-May-71	HB	38.5563	-78.3911
485952	*P. cinereus*	16-May-71	HB	38.5563	-78.3911
485953	*P. cinereus*	16-May-71	HB	38.5563	-78.3911
485954	*P. cinereus*	16-May-71	HB	38.5563	-78.3911
485955	*P. cinereus*	16-May-71	HB	38.5563	-78.3911
485956	*P. cinereus*	16-May-71	HB	38.5563	-78.3911
485957	*P. cinereus*	16-May-71	HB	38.5563	-78.3911
485958	*P. cinereus*	16-May-71	HB	38.5563	-78.3911
485959	*P. cinereus*	16-May-71	HB	38.5563	-78.3911
485960	*P. cinereus*	16-May-71	HB	38.5563	-78.3911
485961	*P. cinereus*	16-May-71	HB	38.5563	-78.3911
485962	*P. cinereus*	16-May-71	HB	38.5563	-78.3911
485963	*P. cinereus*	16-May-71	HB	38.5563	-78.3911
485964	*P. cinereus*	16-May-71	HB	38.5563	-78.3911
485965	*P. cinereus*	16-May-71	HB	38.5563	-78.3911
485966	*P. cinereus*	16-May-71	HB	38.5563	-78.3911
485967	*P. cinereus*	16-May-71	HB	38.5563	-78.3911
485968	*P. cinereus*	16-May-71	HB	38.5563	-78.3911
485969	*P. cinereus*	16-May-71	HB	38.5563	-78.3911
485970	*P. cinereus*	16-May-71	HB	38.5563	-78.3911
485971	*P. cinereus*	16-May-71	HB	38.5563	-78.3911
485972	*P. cinereus*	16-May-71	HB	38.5563	-78.3911
485973	*P. cinereus*	16-May-71	HB	38.5563	-78.3911
485974	*P. cinereus*	16-May-71	HB	38.5563	-78.3911
485975	*P. cinereus*	16-May-71	HB	38.5563	-78.3911
485976	*P. cinereus*	16-May-71	HB	38.5563	-78.3911
485977	*P. cinereus*	16-May-71	HB	38.5563	-78.3911
485978	*P. cinereus*	16-May-71	HB	38.5563	-78.3911
485979	*P. cinereus*	16-May-71	HB	38.5563	-78.3911
485980	*P. cinereus*	16-May-71	HB	38.5563	-78.3911
485981	*P. cinereus*	16-May-71	HB	38.5563	-78.3911
485982	*P. cinereus*	16-May-71	HB	38.5563	-78.3911

485983	*P. cinereus*	16-May-71	HB	38.5563	-78.3911
416060	*P. cinereus*	15-May-72	HB	38.5563	-78.3911
416061	*P. cinereus*	15-May-72	HB	38.5563	-78.3911
416062	*P. cinereus*	15-May-72	HB	38.5563	-78.3911
416063	*P. cinereus*	15-May-72	HB	38.5563	-78.3911
416064	*P. cinereus*	15-May-72	HB	38.5563	-78.3911
416065	*P. cinereus*	15-May-72	HB	38.5563	-78.3911
416068	*P. cinereus*	15-May-72	HB	38.5563	-78.3911
416069	*P. cinereus*	15-May-72	HB	38.5563	-78.3911
416070	*P. cinereus*	15-May-72	HB	38.5563	-78.3911
416072	*P. cinereus*	15-May-72	HB	38.5563	-78.3911
416073	*P. cinereus*	15-May-72	HB	38.5563	-78.3911
416074	*P. cinereus*	15-May-72	HB	38.5563	-78.3911
416041	*P. cinereus*	15-May-72	HB	38.5563	-78.3911
416042	*P. cinereus*	15-May-72	HB	38.5563	-78.3911
416043	*P. cinereus*	15-May-72	HB	38.5563	-78.3911
416044	*P. cinereus*	15-May-72	HB	38.5563	-78.3911
416045	*P. cinereus*	15-May-72	HB	38.5563	-78.3911
416046	*P. cinereus*	15-May-72	HB	38.5563	-78.3911
416047	*P. cinereus*	15-May-72	HB	38.5563	-78.3911
416048	*P. cinereus*	15-May-72	HB	38.5563	-78.3911
416049	*P. cinereus*	15-May-72	HB	38.5563	-78.3911
416050	*P. cinereus*	15-May-72	HB	38.5563	-78.3911
416051	*P. cinereus*	15-May-72	HB	38.5563	-78.3911
416052	*P. cinereus*	15-May-72	HB	38.5563	-78.3911
416053	*P. cinereus*	15-May-72	HB	38.5563	-78.3911
416054	*P. cinereus*	15-May-72	HB	38.5563	-78.3911
416055	*P. cinereus*	15-May-72	HB	38.5563	-78.3911
416056	*P. cinereus*	15-May-72	HB	38.5563	-78.3911
416058	*P. cinereus*	15-May-72	HB	38.5563	-78.3911
416059	*P. cinereus*	15-May-72	HB	38.5563	-78.3911
416084	*P. cinereus*	24-May-73	HB	38.5563	-78.3911
416086	*P. cinereus*	24-May-73	HB	38.5563	-78.3911
416087	*P. cinereus*	24-May-73	HB	38.5563	-78.3911
416088	*P. cinereus*	24-May-73	HB	38.5563	-78.3911
416089	*P. cinereus*	24-May-73	HB	38.5563	-78.3911
416093	*P. cinereus*	27-Jun-79	HB	38.5563	-78.3911
416094	*P. cinereus*	27-Jun-79	HB	38.5563	-78.3911
416095	*P. cinereus*	27-Jun-79	HB	38.5563	-78.3911
416096	*P. cinereus*	27-Jun-79	HB	38.5563	-78.3911
416097	*P. cinereus*	27-Jun-79	HB	38.5563	-78.3911
416098	*P. cinereus*	27-Jun-79	HB	38.5563	-78.3911
416100	*P. cinereus*	27-Jun-79	HB	38.5563	-78.3911
416101	*P. cinereus*	27-Jun-79	HB	38.5563	-78.3911
416102	*P. cinereus*	27-Jun-79	HB	38.5563	-78.3911
416103	*P. cinereus*	27-Jun-79	HB	38.5563	-78.3911
416104	*P. cinereus*	27-Jun-79	HB	38.5563	-78.3911
416105	*P. cinereus*	27-Jun-79	HB	38.5563	-78.3911
416106	*P. cinereus*	27-Jun-79	HB	38.5563	-78.3911

416107	*P. cinereus*	27-Jun-79	HB	38.5563	-78.3911
416108	*P. cinereus*	27-Jun-79	HB	38.5563	-78.3911
416109	*P. cinereus*	27-Jun-79	HB	38.5563	-78.3911
416110	*P. cinereus*	27-Jun-79	HB	38.5563	-78.3911
416111	*P. cinereus*	27-Jun-79	HB	38.5563	-78.3911
416112	*P. cinereus*	27-Jun-79	HB	38.5563	-78.3911
416113	*P. cinereus*	27-Jun-79	HB	38.5563	-78.3911
416114	*P. cinereus*	27-Jun-79	HB	38.5563	-78.3911
416115	*P. cinereus*	27-Jun-79	HB	38.5563	-78.3911
416116	*P. cinereus*	27-Jun-79	HB	38.5563	-78.3911
416117	*P. cinereus*	27-Jun-79	HB	38.5563	-78.3911
416118	*P. cinereus*	27-Jun-79	HB	38.5563	-78.3911
416119	*P. cinereus*	27-Jun-79	HB	38.5563	-78.3911
416120	*P. cinereus*	27-Jun-79	HB	38.5563	-78.3911
416121	*P. cinereus*	27-Jun-79	HB	38.5563	-78.3911
416122	*P. cinereus*	27-Jun-79	HB	38.5563	-78.3911
416123	*P. cinereus*	27-Jun-79	HB	38.5563	-78.3911
416124	*P. cinereus*	27-Jun-79	HB	38.5563	-78.3911
416125	*P. cinereus*	27-Jun-79	HB	38.5563	-78.3911
486062	*P. shenandoah*	15-May-71	HB	38.5563	-78.3911
486063	*P. shenandoah*	15-May-71	HB	38.5563	-78.3911
486064	*P. shenandoah*	15-May-71	HB	38.5563	-78.3911
486065	*P. shenandoah*	15-May-71	HB	38.5563	-78.3911
486066	*P. shenandoah*	15-May-71	HB	38.5563	-78.3911
486067	*P. shenandoah*	15-May-71	HB	38.5563	-78.3911
486068	*P. shenandoah*	15-May-71	HB	38.5563	-78.3911
486069	*P. shenandoah*	15-May-71	HB	38.5563	-78.3911
486070	*P. shenandoah*	15-May-71	HB	38.5563	-78.3911
486071	*P. shenandoah*	15-May-71	HB	38.5563	-78.3911
486072	*P. shenandoah*	15-May-71	HB	38.5563	-78.3911
486073	*P. shenandoah*	15-May-71	HB	38.5563	-78.3911
486074	*P. shenandoah*	15-May-71	HB	38.5563	-78.3911
486075	*P. shenandoah*	15-May-71	HB	38.5563	-78.3911
486076	*P. shenandoah*	15-May-71	HB	38.5563	-78.3911
486077	*P. shenandoah*	15-May-71	HB	38.5563	-78.3911
486078	*P. shenandoah*	15-May-71	HB	38.5563	-78.3911
486079	*P. shenandoah*	15-May-71	HB	38.5563	-78.3911
486080	*P. shenandoah*	15-May-71	HB	38.5563	-78.3911
486081	*P. shenandoah*	15-May-71	HB	38.5563	-78.3911
486082	*P. shenandoah*	15-May-71	HB	38.5563	-78.3911
486083	*P. shenandoah*	15-May-71	HB	38.5563	-78.3911
486084	*P. shenandoah*	15-May-71	HB	38.5563	-78.3911
486085	*P. shenandoah*	15-May-71	HB	38.5563	-78.3911
486086	*P. shenandoah*	15-May-71	HB	38.5563	-78.3911
486087	*P. shenandoah*	15-May-71	HB	38.5563	-78.3911
486088	*P. shenandoah*	15-May-71	HB	38.5563	-78.3911
486089	*P. shenandoah*	15-May-71	HB	38.5563	-78.3911
486090	*P. shenandoah*	15-May-71	HB	38.5563	-78.3911
486091	*P. shenandoah*	15-May-71	HB	38.5563	-78.3911

486092	*P. shenandoah*	15-May-71	HB	38.5563	-78.3911
486093	*P. shenandoah*	15-May-71	HB	38.5563	-78.3911
486094	*P. shenandoah*	15-May-71	HB	38.5563	-78.3911
486095	*P. shenandoah*	15-May-71	HB	38.5563	-78.3911
486096	*P. shenandoah*	15-May-71	HB	38.5563	-78.3911
486097	*P. shenandoah*	15-May-71	HB	38.5563	-78.3911
486098	*P. shenandoah*	15-May-71	HB	38.5563	-78.3911
486099	*P. shenandoah*	15-May-71	HB	38.5563	-78.3911
486100	*P. shenandoah*	15-May-71	HB	38.5563	-78.3911
486101	*P. shenandoah*	15-May-71	HB	38.5563	-78.3911
416077	*P. shenandoah*	24-May-73	HB	38.5563	-78.3911
416078	*P. shenandoah*	24-May-73	HB	38.5563	-78.3911
416079	*P. shenandoah*	24-May-73	HB	38.5563	-78.3911
416080	*P. shenandoah*	24-May-73	HB	38.5563	-78.3911
416081	*P. shenandoah*	24-May-73	HB	38.5563	-78.3911
416082	*P. shenandoah*	24-May-73	HB	38.5563	-78.3911
416083	*P. shenandoah*	24-May-73	HB	38.5563	-78.3911
416090	*P. shenandoah*	27-Jun-79	HB	38.5563	-78.3911
416091	*P. shenandoah*	27-Jun-79	HB	38.5563	-78.3911
416092	*P. shenandoah*	27-Jun-79	HB	38.5563	-78.3911
416126	*P. shenandoah*	27-Jun-79	HB	38.5563	-78.3911
461322	*P. cinereus*	29-Jun-70	IGG	36.11	-82.3611
461323	*P. cinereus*	29-Jun-70	IGG	36.11	-82.3611
461324	*P. cinereus*	29-Jun-70	IGG	36.11	-82.3611
461325	*P. cinereus*	29-Jun-70	IGG	36.11	-82.3611
461326	*P. cinereus*	29-Jun-70	IGG	36.11	-82.3611
461327	*P. cinereus*	29-Jun-70	IGG	36.11	-82.3611
461409	*P. cinereus*	28-May-72	IGG	36.11	-82.3611
461396	*P. cinereus*	28-May-72	IGG	36.11	-82.3611
461397	*P. cinereus*	28-May-72	IGG	36.11	-82.3611
461398	*P. cinereus*	28-May-72	IGG	36.11	-82.3611
461399	*P. cinereus*	28-May-72	IGG	36.11	-82.3611
461400	*P. cinereus*	28-May-72	IGG	36.11	-82.3611
461401	*P. cinereus*	28-May-72	IGG	36.11	-82.3611
461402	*P. cinereus*	28-May-72	IGG	36.11	-82.3611
461403	*P. cinereus*	28-May-72	IGG	36.11	-82.3611
461404	*P. cinereus*	28-May-72	IGG	36.11	-82.3611
461405	*P. cinereus*	28-May-72	IGG	36.11	-82.3611
461406	*P. cinereus*	28-May-72	IGG	36.11	-82.3611
461407	*P. cinereus*	28-May-72	IGG	36.11	-82.3611
461408	*P. cinereus*	28-May-72	IGG	36.11	-82.3611
461410	*P. cinereus*	28-May-72	IGG	36.11	-82.3611
461411	*P. cinereus*	28-May-72	IGG	36.11	-82.3611
461412	*P. cinereus*	28-May-72	IGG	36.11	-82.3611
461413	*P. cinereus*	28-May-72	IGG	36.11	-82.3611
461414	*P. cinereus*	28-May-72	IGG	36.11	-82.3611
461415	*P. cinereus*	28-May-72	IGG	36.11	-82.3611
461487	*P. cinereus*	6-Mar-76	IGG	36.11	-82.3611
461488	*P. cinereus*	6-Mar-76	IGG	36.11	-82.3611

461489	*P. cinereus*	6-Mar-76	IGG	36.11	-82.3611
461490	*P. cinereus*	6-Mar-76	IGG	36.11	-82.3611
461491	*P. cinereus*	6-Mar-76	IGG	36.11	-82.3611
461492	*P. cinereus*	6-Mar-76	IGG	36.11	-82.3611
461493	*P. cinereus*	6-Mar-76	IGG	36.11	-82.3611
461494	*P. cinereus*	6-Mar-76	IGG	36.11	-82.3611
461495	*P. cinereus*	6-Mar-76	IGG	36.11	-82.3611
461496	*P. cinereus*	6-Mar-76	IGG	36.11	-82.3611
461497	*P. cinereus*	6-Mar-76	IGG	36.11	-82.3611
461498	*P. cinereus*	6-Mar-76	IGG	36.11	-82.3611
461499	*P. cinereus*	6-Mar-76	IGG	36.11	-82.3611
461500	*P. cinereus*	6-Mar-76	IGG	36.11	-82.3611
461503	*P. cinereus*	6-Mar-76	IGG	36.11	-82.3611
461504	*P. cinereus*	6-Mar-76	IGG	36.11	-82.3611
461505	*P. cinereus*	6-Mar-76	IGG	36.11	-82.3611
461506	*P. cinereus*	6-Mar-76	IGG	36.11	-82.3611
461507	*P. cinereus*	6-Mar-76	IGG	36.11	-82.3611
461508	*P. cinereus*	6-Mar-76	IGG	36.11	-82.3611
461509	*P. cinereus*	6-Mar-76	IGG	36.11	-82.3611
461510	*P. cinereus*	6-Mar-76	IGG	36.11	-82.3611
461511	*P. cinereus*	6-Mar-76	IGG	36.11	-82.3611
461512	*P. cinereus*	6-Mar-76	IGG	36.11	-82.3611
461513	*P. cinereus*	6-Mar-76	IGG	36.11	-82.3611
461514	*P. cinereus*	6-Mar-76	IGG	36.11	-82.3611
461552	*P. cinereus*	6-Mar-76	IGG	36.11	-82.3611
461553	*P. cinereus*	6-Mar-76	IGG	36.11	-82.3611
461608	*P. cinereus*	15-May-76	IGG	36.11	-82.3611
461609	*P. cinereus*	15-May-76	IGG	36.11	-82.3611
461610	*P. cinereus*	15-May-76	IGG	36.11	-82.3611
461611	*P. cinereus*	15-May-76	IGG	36.11	-82.3611
461612	*P. cinereus*	15-May-76	IGG	36.11	-82.3611
461613	*P. cinereus*	15-May-76	IGG	36.11	-82.3611
461614	*P. cinereus*	15-May-76	IGG	36.11	-82.3611
461615	*P. cinereus*	15-May-76	IGG	36.11	-82.3611
461616	*P. cinereus*	15-May-76	IGG	36.11	-82.3611
461618	*P. cinereus*	15-May-76	IGG	36.11	-82.3611
461619	*P. cinereus*	15-May-76	IGG	36.11	-82.3611
461620	*P. cinereus*	15-May-76	IGG	36.11	-82.3611
461621	*P. cinereus*	15-May-76	IGG	36.11	-82.3611
461622	*P. cinereus*	15-May-76	IGG	36.11	-82.3611
461623	*P. cinereus*	15-May-76	IGG	36.11	-82.3611
461624	*P. cinereus*	15-May-76	IGG	36.11	-82.3611
461625	*P. cinereus*	15-May-76	IGG	36.11	-82.3611
461665	*P. cinereus*	15-Sep-76	IGG	36.11	-82.3611
461666	*P. cinereus*	15-Sep-76	IGG	36.11	-82.3611
461667	*P. cinereus*	15-Sep-76	IGG	36.11	-82.3611
461668	*P. cinereus*	15-Sep-76	IGG	36.11	-82.3611
461669	*P. cinereus*	15-Sep-76	IGG	36.11	-82.3611
461670	*P. cinereus*	15-Sep-76	IGG	36.11	-82.3611

461671	*P. cinereus*	15-Sep-76	IGG	36.11	-82.3611
461672	*P. cinereus*	15-Sep-76	IGG	36.11	-82.3611
461673	*P. cinereus*	15-Sep-76	IGG	36.11	-82.3611
461674	*P. cinereus*	15-Sep-76	IGG	36.11	-82.3611
461675	*P. cinereus*	15-Sep-76	IGG	36.11	-82.3611
461676	*P. cinereus*	15-Sep-76	IGG	36.11	-82.3611
461677	*P. cinereus*	15-Sep-76	IGG	36.11	-82.3611
461678	*P. cinereus*	15-Sep-76	IGG	36.11	-82.3611
461679	*P. cinereus*	15-Sep-76	IGG	36.11	-82.3611
461680	*P. cinereus*	15-Sep-76	IGG	36.11	-82.3611
461681	*P. cinereus*	15-Sep-76	IGG	36.11	-82.3611
461682	*P. cinereus*	15-Sep-76	IGG	36.11	-82.3611
461683	*P. cinereus*	15-Sep-76	IGG	36.11	-82.3611
461684	*P. cinereus*	15-Sep-76	IGG	36.11	-82.3611
461685	*P. cinereus*	15-Sep-76	IGG	36.11	-82.3611
461809	*P. cinereus*	15-Aug-77	IGG	36.11	-82.3611
461810	*P. cinereus*	15-Aug-77	IGG	36.11	-82.3611
461811	*P. cinereus*	15-Aug-77	IGG	36.11	-82.3611
461812	*P. cinereus*	15-Aug-77	IGG	36.11	-82.3611
461813	*P. cinereus*	15-Aug-77	IGG	36.11	-82.3611
461814	*P. cinereus*	15-Aug-77	IGG	36.11	-82.3611
461815	*P. cinereus*	15-Aug-77	IGG	36.11	-82.3611
461816	*P. cinereus*	15-Aug-77	IGG	36.11	-82.3611
461817	*P. cinereus*	15-Aug-77	IGG	36.11	-82.3611
461818	*P. cinereus*	15-Aug-77	IGG	36.11	-82.3611
461819	*P. cinereus*	15-Aug-77	IGG	36.11	-82.3611
461885	*P. cinereus*	24-Aug-78	IGG	36.11	-82.3611
461886	*P. cinereus*	24-Aug-78	IGG	36.11	-82.3611
461887	*P. cinereus*	24-Aug-78	IGG	36.11	-82.3611
461888	*P. cinereus*	24-Aug-78	IGG	36.11	-82.3611
461889	*P. cinereus*	24-Aug-78	IGG	36.11	-82.3611
461890	*P. cinereus*	24-Aug-78	IGG	36.11	-82.3611
461891	*P. cinereus*	24-Aug-78	IGG	36.11	-82.3611
461893	*P. cinereus*	24-Aug-78	IGG	36.11	-82.3611
461895	*P. cinereus*	24-Aug-78	IGG	36.11	-82.3611
461896	*P. cinereus*	24-Aug-78	IGG	36.11	-82.3611
461897	*P. cinereus*	24-Aug-78	IGG	36.11	-82.3611
461898	*P. cinereus*	24-Aug-78	IGG	36.11	-82.3611
461915	*P. cinereus*	24-Aug-78	IGG	36.11	-82.3611
461932	*P. cinereus*	21-Oct-79	IGG	36.11	-82.3611
461933	*P. cinereus*	21-Oct-79	IGG	36.11	-82.3611
461934	*P. cinereus*	21-Oct-79	IGG	36.11	-82.3611
461935	*P. cinereus*	21-Oct-79	IGG	36.11	-82.3611
461936	*P. cinereus*	21-Oct-79	IGG	36.11	-82.3611
461937	*P. cinereus*	21-Oct-79	IGG	36.11	-82.3611
461938	*P. cinereus*	21-Oct-79	IGG	36.11	-82.3611
461940	*P. cinereus*	21-Oct-79	IGG	36.11	-82.3611
461941	*P. cinereus*	21-Oct-79	IGG	36.11	-82.3611
462029	*P. cinereus*	15-Jul-84	IGG	36.11	-82.3611

462030	*P. cinereus*	15-Jul-84	IGG	36.11	-82.3611
462031	*P. cinereus*	15-Jul-84	IGG	36.11	-82.3611
462032	*P. cinereus*	15-Jul-84	IGG	36.11	-82.3611
462033	*P. cinereus*	15-Jul-84	IGG	36.11	-82.3611
462034	*P. cinereus*	15-Jul-84	IGG	36.11	-82.3611
462035	*P. cinereus*	15-Jul-84	IGG	36.11	-82.3611
462036	*P. cinereus*	15-Jul-84	IGG	36.11	-82.3611
462037	*P. cinereus*	15-Jul-84	IGG	36.11	-82.3611
462038	*P. cinereus*	15-Jul-84	IGG	36.11	-82.3611
462039	*P. cinereus*	15-Jul-84	IGG	36.11	-82.3611
462040	*P. cinereus*	15-Jul-84	IGG	36.11	-82.3611
462059	*P. cinereus*	24-Mar-85	IGG	36.11	-82.3611
462060	*P. cinereus*	24-Mar-85	IGG	36.11	-82.3611
462061	*P. cinereus*	24-Mar-85	IGG	36.11	-82.3611
462062	*P. cinereus*	24-Mar-85	IGG	36.11	-82.3611
462063	*P. cinereus*	24-Mar-85	IGG	36.11	-82.3611
462064	*P. cinereus*	24-Mar-85	IGG	36.11	-82.3611
462065	*P. cinereus*	24-Mar-85	IGG	36.11	-82.3611
462066	*P. cinereus*	24-Mar-85	IGG	36.11	-82.3611
462067	*P. cinereus*	24-Mar-85	IGG	36.11	-82.3611
462069	*P. cinereus*	24-Mar-85	IGG	36.11	-82.3611
462071	*P. cinereus*	24-Mar-85	IGG	36.11	-82.3611
462072	*P. cinereus*	24-Mar-85	IGG	36.11	-82.3611
462073	*P. cinereus*	24-Mar-85	IGG	36.11	-82.3611
462074	*P. cinereus*	24-Mar-85	IGG	36.11	-82.3611
462075	*P. cinereus*	24-Mar-85	IGG	36.11	-82.3611
462076	*P. cinereus*	24-Mar-85	IGG	36.11	-82.3611
462077	*P. cinereus*	24-Mar-85	IGG	36.11	-82.3611
462078	*P. cinereus*	24-Mar-85	IGG	36.11	-82.3611
462079	*P. cinereus*	24-Mar-85	IGG	36.11	-82.3611
462080	*P. cinereus*	24-Mar-85	IGG	36.11	-82.3611
462118	*P. cinereus*	25-Apr-86	IGG	36.11	-82.3611
462119	*P. cinereus*	25-Apr-86	IGG	36.11	-82.3611
462120	*P. cinereus*	25-Apr-86	IGG	36.11	-82.3611
462121	*P. cinereus*	25-Apr-86	IGG	36.11	-82.3611
462129	*P. cinereus*	26-Apr-86	IGG	36.11	-82.3611
462130	*P. cinereus*	26-Apr-86	IGG	36.11	-82.3611
462131	*P. cinereus*	26-Apr-86	IGG	36.11	-82.3611
462132	*P. cinereus*	26-Apr-86	IGG	36.11	-82.3611
462133	*P. cinereus*	26-Apr-86	IGG	36.11	-82.3611
462135	*P. cinereus*	26-Apr-86	IGG	36.11	-82.3611
462136	*P. cinereus*	26-Apr-86	IGG	36.11	-82.3611
462137	*P. cinereus*	26-Apr-86	IGG	36.11	-82.3611
462138	*P. cinereus*	26-Apr-86	IGG	36.11	-82.3611
462140	*P. cinereus*	26-Apr-86	IGG	36.11	-82.3611
462141	*P. cinereus*	26-Apr-86	IGG	36.11	-82.3611
462142	*P. cinereus*	26-Apr-86	IGG	36.11	-82.3611
462144	*P. cinereus*	26-Apr-86	IGG	36.11	-82.3611
462146	*P. cinereus*	26-Apr-86	IGG	36.11	-82.3611

462148	*P. cinereus*	26-Apr-86	IGG	36.11	-82.3611
462149	*P. cinereus*	26-Apr-86	IGG	36.11	-82.3611
462185	*P. cinereus*	23-Jun-87	IGG	36.11	-82.3611
462186	*P. cinereus*	23-Jun-87	IGG	36.11	-82.3611
462187	*P. cinereus*	23-Jun-87	IGG	36.11	-82.3611
462190	*P. cinereus*	23-Jun-87	IGG	36.11	-82.3611
461307	*P. cylindraceus*	29-Jun-70	IGG	36.11	-82.3611
461312	*P. cylindraceus*	29-Jun-70	IGG	36.11	-82.3611
461313	*P. cylindraceus*	29-Jun-70	IGG	36.11	-82.3611
461317	*P. cylindraceus*	29-Jun-70	IGG	36.11	-82.3611
461321	*P. cylindraceus*	29-Jun-70	IGG	36.11	-82.3611
461336	*P. cylindraceus*	28-May-72	IGG	36.11	-82.3611
461337	*P. cylindraceus*	28-May-72	IGG	36.11	-82.3611
461338	*P. cylindraceus*	28-May-72	IGG	36.11	-82.3611
461339	*P. cylindraceus*	28-May-72	IGG	36.11	-82.3611
461340	*P. cylindraceus*	28-May-72	IGG	36.11	-82.3611
461341	*P. cylindraceus*	28-May-72	IGG	36.11	-82.3611
461342	*P. cylindraceus*	28-May-72	IGG	36.11	-82.3611
461343	*P. cylindraceus*	28-May-72	IGG	36.11	-82.3611
461344	*P. cylindraceus*	28-May-72	IGG	36.11	-82.3611
461345	*P. cylindraceus*	28-May-72	IGG	36.11	-82.3611
461346	*P. cylindraceus*	28-May-72	IGG	36.11	-82.3611
461347	*P. cylindraceus*	28-May-72	IGG	36.11	-82.3611
461348	*P. cylindraceus*	28-May-72	IGG	36.11	-82.3611
461349	*P. cylindraceus*	28-May-72	IGG	36.11	-82.3611
461357	*P. cylindraceus*	28-May-72	IGG	36.11	-82.3611
461358	*P. cylindraceus*	28-May-72	IGG	36.11	-82.3611
461359	*P. cylindraceus*	28-May-72	IGG	36.11	-82.3611
461360	*P. cylindraceus*	28-May-72	IGG	36.11	-82.3611
461361	*P. cylindraceus*	28-May-72	IGG	36.11	-82.3611
461362	*P. cylindraceus*	28-May-72	IGG	36.11	-82.3611
461363	*P. cylindraceus*	28-May-72	IGG	36.11	-82.3611
461364	*P. cylindraceus*	28-May-72	IGG	36.11	-82.3611
461365	*P. cylindraceus*	28-May-72	IGG	36.11	-82.3611
461366	*P. cylindraceus*	28-May-72	IGG	36.11	-82.3611
461367	*P. cylindraceus*	28-May-72	IGG	36.11	-82.3611
461421	*P. cylindraceus*	21-May-73	IGG	36.11	-82.3611
461422	*P. cylindraceus*	21-May-73	IGG	36.11	-82.3611
461423	*P. cylindraceus*	21-May-73	IGG	36.11	-82.3611
461424	*P. cylindraceus*	21-May-73	IGG	36.11	-82.3611
461425	*P. cylindraceus*	21-May-73	IGG	36.11	-82.3611
461426	*P. cylindraceus*	21-May-73	IGG	36.11	-82.3611
461427	*P. cylindraceus*	21-May-73	IGG	36.11	-82.3611
461428	*P. cylindraceus*	21-May-73	IGG	36.11	-82.3611
461429	*P. cylindraceus*	21-May-73	IGG	36.11	-82.3611
461430	*P. cylindraceus*	21-May-73	IGG	36.11	-82.3611
461431	*P. cylindraceus*	21-May-73	IGG	36.11	-82.3611
461432	*P. cylindraceus*	21-May-73	IGG	36.11	-82.3611
461473	*P. cylindraceus*	4-Jul-75	IGG	36.11	-82.3611

461474	*P. cylindraceus*	4-Jul-75	IGG	36.11	-82.3611
461475	*P. cylindraceus*	4-Jul-75	IGG	36.11	-82.3611
461476	*P. cylindraceus*	4-Jul-75	IGG	36.11	-82.3611
461477	*P. cylindraceus*	4-Jul-75	IGG	36.11	-82.3611
461478	*P. cylindraceus*	4-Jul-75	IGG	36.11	-82.3611
461566	*P. cylindraceus*	15-May-76	IGG	36.11	-82.3611
461567	*P. cylindraceus*	15-May-76	IGG	36.11	-82.3611
461568	*P. cylindraceus*	15-May-76	IGG	36.11	-82.3611
461569	*P. cylindraceus*	15-May-76	IGG	36.11	-82.3611
461570	*P. cylindraceus*	15-May-76	IGG	36.11	-82.3611
461571	*P. cylindraceus*	15-May-76	IGG	36.11	-82.3611
461572	*P. cylindraceus*	15-May-76	IGG	36.11	-82.3611
461573	*P. cylindraceus*	15-May-76	IGG	36.11	-82.3611
461574	*P. cylindraceus*	15-May-76	IGG	36.11	-82.3611
461575	*P. cylindraceus*	15-May-76	IGG	36.11	-82.3611
461576	*P. cylindraceus*	15-May-76	IGG	36.11	-82.3611
461577	*P. cylindraceus*	15-May-76	IGG	36.11	-82.3611
461578	*P. cylindraceus*	15-May-76	IGG	36.11	-82.3611
461579	*P. cylindraceus*	15-May-76	IGG	36.11	-82.3611
461580	*P. cylindraceus*	15-May-76	IGG	36.11	-82.3611
461581	*P. cylindraceus*	15-May-76	IGG	36.11	-82.3611
461582	*P. cylindraceus*	15-May-76	IGG	36.11	-82.3611
461583	*P. cylindraceus*	15-May-76	IGG	36.11	-82.3611
461584	*P. cylindraceus*	15-May-76	IGG	36.11	-82.3611
461585	*P. cylindraceus*	15-May-76	IGG	36.11	-82.3611
461586	*P. cylindraceus*	15-May-76	IGG	36.11	-82.3611
461587	*P. cylindraceus*	15-May-76	IGG	36.11	-82.3611
461749	*P. cylindraceus*	15-Sep-76	IGG	36.11	-82.3611
461750	*P. cylindraceus*	15-Sep-76	IGG	36.11	-82.3611
461754	*P. cylindraceus*	15-Sep-76	IGG	36.11	-82.3611
461755	*P. cylindraceus*	15-Sep-76	IGG	36.11	-82.3611
461758	*P. cylindraceus*	15-Sep-76	IGG	36.11	-82.3611
461759	*P. cylindraceus*	15-Sep-76	IGG	36.11	-82.3611
461760	*P. cylindraceus*	15-Sep-76	IGG	36.11	-82.3611
461761	*P. cylindraceus*	15-Sep-76	IGG	36.11	-82.3611
461762	*P. cylindraceus*	15-Sep-76	IGG	36.11	-82.3611
461764	*P. cylindraceus*	15-Sep-76	IGG	36.11	-82.3611
461765	*P. cylindraceus*	15-Sep-76	IGG	36.11	-82.3611
461767	*P. cylindraceus*	15-Sep-76	IGG	36.11	-82.3611
461768	*P. cylindraceus*	15-Sep-76	IGG	36.11	-82.3611
461769	*P. cylindraceus*	15-Sep-76	IGG	36.11	-82.3611
461770	*P. cylindraceus*	15-Sep-76	IGG	36.11	-82.3611
461771	*P. cylindraceus*	15-Sep-76	IGG	36.11	-82.3611
461772	*P. cylindraceus*	15-Sep-76	IGG	36.11	-82.3611
461773	*P. cylindraceus*	15-Sep-76	IGG	36.11	-82.3611
461774	*P. cylindraceus*	15-Sep-76	IGG	36.11	-82.3611
461776	*P. cylindraceus*	15-Sep-76	IGG	36.11	-82.3611
461737	*P. cylindraceus*	14-Aug-77	IGG	36.11	-82.3611
461738	*P. cylindraceus*	14-Aug-77	IGG	36.11	-82.3611

461739	*P. cylindraceus*	14-Aug-77	IGG	36.11	-82.3611
461740	*P. cylindraceus*	14-Aug-77	IGG	36.11	-82.3611
461742	*P. cylindraceus*	14-Aug-77	IGG	36.11	-82.3611
461743	*P. cylindraceus*	14-Aug-77	IGG	36.11	-82.3611
461744	*P. cylindraceus*	14-Aug-77	IGG	36.11	-82.3611
461745	*P. cylindraceus*	14-Aug-77	IGG	36.11	-82.3611
461746	*P. cylindraceus*	14-Aug-77	IGG	36.11	-82.3611
461783	*P. cylindraceus*	15-Aug-77	IGG	36.11	-82.3611
461784	*P. cylindraceus*	15-Aug-77	IGG	36.11	-82.3611
461785	*P. cylindraceus*	15-Aug-77	IGG	36.11	-82.3611
461786	*P. cylindraceus*	15-Aug-77	IGG	36.11	-82.3611
461787	*P. cylindraceus*	15-Aug-77	IGG	36.11	-82.3611
461788	*P. cylindraceus*	15-Aug-77	IGG	36.11	-82.3611
461789	*P. cylindraceus*	15-Aug-77	IGG	36.11	-82.3611
461790	*P. cylindraceus*	15-Aug-77	IGG	36.11	-82.3611
461791	*P. cylindraceus*	15-Aug-77	IGG	36.11	-82.3611
461792	*P. cylindraceus*	15-Aug-77	IGG	36.11	-82.3611
461793	*P. cylindraceus*	15-Aug-77	IGG	36.11	-82.3611
461794	*P. cylindraceus*	15-Aug-77	IGG	36.11	-82.3611
461796	*P. cylindraceus*	15-Aug-77	IGG	36.11	-82.3611
461797	*P. cylindraceus*	15-Aug-77	IGG	36.11	-82.3611
461798	*P. cylindraceus*	15-Aug-77	IGG	36.11	-82.3611
461799	*P. cylindraceus*	15-Aug-77	IGG	36.11	-82.3611
461800	*P. cylindraceus*	15-Aug-77	IGG	36.11	-82.3611
461801	*P. cylindraceus*	15-Aug-77	IGG	36.11	-82.3611
461802	*P. cylindraceus*	15-Aug-77	IGG	36.11	-82.3611
461803	*P. cylindraceus*	15-Aug-77	IGG	36.11	-82.3611
461804	*P. cylindraceus*	15-Aug-77	IGG	36.11	-82.3611
461807	*P. cylindraceus*	15-Aug-77	IGG	36.11	-82.3611
461808	*P. cylindraceus*	15-Aug-77	IGG	36.11	-82.3611
461873	*P. cylindraceus*	24-Aug-78	IGG	36.11	-82.3611
461874	*P. cylindraceus*	24-Aug-78	IGG	36.11	-82.3611
461875	*P. cylindraceus*	24-Aug-78	IGG	36.11	-82.3611
461876	*P. cylindraceus*	24-Aug-78	IGG	36.11	-82.3611
461877	*P. cylindraceus*	24-Aug-78	IGG	36.11	-82.3611
461878	*P. cylindraceus*	24-Aug-78	IGG	36.11	-82.3611
461879	*P. cylindraceus*	24-Aug-78	IGG	36.11	-82.3611
461880	*P. cylindraceus*	24-Aug-78	IGG	36.11	-82.3611
461881	*P. cylindraceus*	24-Aug-78	IGG	36.11	-82.3611
461882	*P. cylindraceus*	24-Aug-78	IGG	36.11	-82.3611
461883	*P. cylindraceus*	24-Aug-78	IGG	36.11	-82.3611
461884	*P. cylindraceus*	24-Aug-78	IGG	36.11	-82.3611
461917	*P. cylindraceus*	21-Oct-79	IGG	36.11	-82.3611
461918	*P. cylindraceus*	21-Oct-79	IGG	36.11	-82.3611
461919	*P. cylindraceus*	21-Oct-79	IGG	36.11	-82.3611
461920	*P. cylindraceus*	21-Oct-79	IGG	36.11	-82.3611
461921	*P. cylindraceus*	21-Oct-79	IGG	36.11	-82.3611
461922	*P. cylindraceus*	21-Oct-79	IGG	36.11	-82.3611
461923	*P. cylindraceus*	21-Oct-79	IGG	36.11	-82.3611

461924	*P. cylindraceus*	21-Oct-79	IGG	36.11	-82.3611
461928	*P. cylindraceus*	21-Oct-79	IGG	36.11	-82.3611
462007	*P. cylindraceus*	10-Jun-84	IGG	36.11	-82.3611
462008	*P. cylindraceus*	10-Jun-84	IGG	36.11	-82.3611
462009	*P. cylindraceus*	10-Jun-84	IGG	36.11	-82.3611
462024	*P. cylindraceus*	15-Jul-84	IGG	36.11	-82.3611
462025	*P. cylindraceus*	15-Jul-84	IGG	36.11	-82.3611
462026	*P. cylindraceus*	15-Jul-84	IGG	36.11	-82.3611
462027	*P. cylindraceus*	15-Jul-84	IGG	36.11	-82.3611
462058	*P. cylindraceus*	24-Mar-85	IGG	36.11	-82.3611
462126	*P. cylindraceus*	26-Apr-86	IGG	36.11	-82.3611
462127	*P. cylindraceus*	26-Apr-86	IGG	36.11	-82.3611
462128	*P. cylindraceus*	26-Apr-86	IGG	36.11	-82.3611
462180	*P. cylindraceus*	23-Jun-87	IGG	36.11	-82.3611
462181	*P. cylindraceus*	23-Jun-87	IGG	36.11	-82.3611
462182	*P. cylindraceus*	23-Jun-87	IGG	36.11	-82.3611
462183	*P. cylindraceus*	23-Jun-87	IGG	36.11	-82.3611
424021	*P. cinereus*	9-Jun-78	RIM	36.6653	-81.7025
424022	*P. cinereus*	9-Jun-78	RIM	36.6653	-81.7025
424023	*P. cinereus*	9-Jun-78	RIM	36.6653	-81.7025
424075	*P. cinereus*	12-Oct-78	RIM	36.6653	-81.7025
424076	*P. cinereus*	12-Oct-78	RIM	36.6653	-81.7025
424077	*P. cinereus*	12-Oct-78	RIM	36.6653	-81.7025
424078	*P. cinereus*	12-Oct-78	RIM	36.6653	-81.7025
423957	*P. cinereus*	20-May-72	RIM	36.6653	-81.7025
423958	*P. cinereus*	20-May-72	RIM	36.6653	-81.7025
423997	*P. cinereus*	24-Mar-78	RIM	36.6653	-81.7025
423998	*P. cinereus*	24-Mar-78	RIM	36.6653	-81.7025
423999	*P. cinereus*	24-Mar-78	RIM	36.6653	-81.7025
424000	*P. cinereus*	24-Mar-78	RIM	36.6653	-81.7025
424095	*P. cylindraceus*	10-Jun-81	RIM	36.6653	-81.7025
424096	*P. cylindraceus*	10-Jun-81	RIM	36.6653	-81.7025
424097	*P. cylindraceus*	10-Jun-81	RIM	36.6653	-81.7025
423965	*P. cylindraceus*	20-May-72	RIM	36.6653	-81.7025
423967	*P. cylindraceus*	20-May-72	RIM	36.6653	-81.7025
423969	*P. cylindraceus*	20-May-72	RIM	36.6653	-81.7025
423972	*P. cylindraceus*	20-May-72	RIM	36.6653	-81.7025
423973	*P. cylindraceus*	20-May-72	RIM	36.6653	-81.7025
423974	*P. cylindraceus*	20-May-72	RIM	36.6653	-81.7025
423975	*P. cylindraceus*	20-May-72	RIM	36.6653	-81.7025
423976	*P. cylindraceus*	20-May-72	RIM	36.6653	-81.7025
423979	*P. cylindraceus*	20-May-72	RIM	36.6653	-81.7025
486354	*P. cylindraceus*	2-Jun-87	RIM	36.6653	-81.7025
486355	*P. cylindraceus*	2-Jun-87	RIM	36.6653	-81.7025
486356	*P. cylindraceus*	2-Jun-87	RIM	36.6653	-81.7025
486358	*P. cylindraceus*	2-Jun-87	RIM	36.6653	-81.7025
486350	*P. cylindraceus*	27-May-87	RIM	36.6653	-81.7025
424028	*P. richmondi*	9-Jun-78	RIM	36.6653	-81.7025
424029	*P. richmondi*	9-Jun-78	RIM	36.6653	-81.7025

424030	*P. richmondi*	9-Jun-78	RIM	36.6653	-81.7025
424032	*P. richmondi*	9-Jun-78	RIM	36.6653	-81.7025
424033	*P. richmondi*	9-Jun-78	RIM	36.6653	-81.7025
424034	*P. richmondi*	9-Jun-78	RIM	36.6653	-81.7025
424035	*P. richmondi*	9-Jun-78	RIM	36.6653	-81.7025
424036	*P. richmondi*	9-Jun-78	RIM	36.6653	-81.7025
424037	*P. richmondi*	9-Jun-78	RIM	36.6653	-81.7025
424038	*P. richmondi*	9-Jun-78	RIM	36.6653	-81.7025
424039	*P. richmondi*	9-Jun-78	RIM	36.6653	-81.7025
424040	*P. richmondi*	9-Jun-78	RIM	36.6653	-81.7025
424041	*P. richmondi*	9-Jun-78	RIM	36.6653	-81.7025
424042	*P. richmondi*	9-Jun-78	RIM	36.6653	-81.7025
424043	*P. richmondi*	9-Jun-78	RIM	36.6653	-81.7025
424100	*P. richmondi*	10-Jun-81	RIM	36.6653	-81.7025
423987	*P. richmondi*	14-Aug-77	RIM	36.6653	-81.7025
423955	*P. richmondi*	20-May-72	RIM	36.6653	-81.7025
423956	*P. richmondi*	20-May-72	RIM	36.6653	-81.7025
424005	*P. richmondi*	24-Mar-78	RIM	36.6653	-81.7025
424006	*P. richmondi*	24-Mar-78	RIM	36.6653	-81.7025
424007	*P. richmondi*	24-Mar-78	RIM	36.6653	-81.7025
424008	*P. richmondi*	24-Mar-78	RIM	36.6653	-81.7025
424010	*P. richmondi*	24-Mar-78	RIM	36.6653	-81.7025
424011	*P. richmondi*	24-Mar-78	RIM	36.6653	-81.7025
424012	*P. richmondi*	24-Mar-78	RIM	36.6653	-81.7025
424013	*P. richmondi*	24-Mar-78	RIM	36.6653	-81.7025
424014	*P. richmondi*	24-Mar-78	RIM	36.6653	-81.7025
424015	*P. richmondi*	24-Mar-78	RIM	36.6653	-81.7025
424016	*P. richmondi*	24-Mar-78	RIM	36.6653	-81.7025
424017	*P. richmondi*	24-Mar-78	RIM	36.6653	-81.7025
474858	*P. glutinosus*	2-Oct-71	WOS	35.63889	-83.7578
474860	*P. glutinosus*	2-Oct-71	WOS	35.63889	-83.7578
474861	*P. glutinosus*	2-Oct-71	WOS	35.63889	-83.7578
474862	*P. glutinosus*	2-Oct-71	WOS	35.63889	-83.7578
474864	*P. glutinosus*	2-Oct-71	WOS	35.63889	-83.7578
474865	*P. glutinosus*	2-Oct-71	WOS	35.63889	-83.7578
474866	*P. glutinosus*	2-Oct-71	WOS	35.63889	-83.7578
474867	*P. glutinosus*	2-Oct-71	WOS	35.63889	-83.7578
474868	*P. glutinosus*	2-Oct-71	WOS	35.63889	-83.7578
474869	*P. glutinosus*	2-Oct-71	WOS	35.63889	-83.7578
474870	*P. glutinosus*	2-Oct-71	WOS	35.63889	-83.7578
474871	*P. glutinosus*	9-Mar-74	WOS	35.63889	-83.7578
474872	*P. glutinosus*	9-Mar-74	WOS	35.63889	-83.7578
474873	*P. glutinosus*	9-Mar-74	WOS	35.63889	-83.7578
474874	*P. glutinosus*	9-Mar-74	WOS	35.63889	-83.7578
474875	*P. glutinosus*	9-Mar-74	WOS	35.63889	-83.7578
474876	*P. glutinosus*	9-Mar-74	WOS	35.63889	-83.7578
474877	*P. glutinosus*	9-Mar-74	WOS	35.63889	-83.7578
474878	*P. glutinosus*	9-Mar-74	WOS	35.63889	-83.7578
474879	*P. glutinosus*	9-Mar-74	WOS	35.63889	-83.7578

474880	*P. glutinosus*	9-Mar-74	WOS	35.63889	-83.7578
474881	*P. glutinosus*	9-Mar-74	WOS	35.63889	-83.7578
474882	*P. glutinosus*	9-Mar-74	WOS	35.63889	-83.7578
474884	*P. glutinosus*	26-Apr-74	WOS	35.63889	-83.7578
474887	*P. glutinosus*	26-Apr-74	WOS	35.63889	-83.7578
474888	*P. glutinosus*	26-Apr-74	WOS	35.63889	-83.7578
474890	*P. glutinosus*	26-Apr-74	WOS	35.63889	-83.7578
474891	*P. glutinosus*	26-Apr-74	WOS	35.63889	-83.7578
466008	*P. serratus*	9-Mar-74	WOS	35.63889	-83.7578
466009	*P. serratus*	9-Mar-74	WOS	35.63889	-83.7578
466036	*P. serratus*	26-Apr-74	WOS	35.63889	-83.7578
